# Walking-Related Dual-Task Interference in Early-to-Middle-Stage Huntington's Disease: An Auditory Event Related Potential Study

**DOI:** 10.3389/fpsyg.2017.01292

**Published:** 2017-07-31

**Authors:** Marina de Tommaso, Katia Ricci, Anna Montemurno, Eleonora Vecchio, Sara Invitto

**Affiliations:** ^1^Neurophysiopathology of Pain, Basic Medical Science, Neuroscience and Sensory System Department–SMBNOS-Bari Aldo Moro University Bari, Italy; ^2^Department of Biological and Environmental Sciences and Technologies, University of Salento Lecce, Italy

**Keywords:** dual task, acoustic paradigm, P3, walking, Huntington's disease

## Abstract

**Objective:** To compare interference between walking and a simple P3 auditory odd-ball paradigm in patients with Huntington's disease (HD) and age- and sex-matched controls.

**Methods:** Twenty-four early-to-middle-stage HD patients and 14 age- and sex-matched healthy volunteers were examined. EEG—EMG recordings were obtained from 21 scalp electrodes and eight bipolar derivations from the legs. Principal component analysis was used to obtain artifact-free recordings. The stimulation paradigm consisted of 50 rare and 150 frequent stimuli and was performed in two conditions: standing and walking along a 10 by 5 m path. P3 wave amplitude and latency and EEG and EMG spectral values were compared by group and experimental condition and correlated with clinical features of HD.

**Results:** P3 amplitude increased during walking in both HD patients and controls. This effect was inversely correlated with motor impairment in HD patients, who showed a beta-band power increase over the parieto-occipital regions in the walking condition during the P3 task. Walking speed and counting of rare stimuli were not compromised by concurrence of motor and cognitive demands.

**Conclusion:** Our results showed that walking increased P3 amplitude in an auditory task, in both HD patients and controls. Concurrent cognitive and motor stimulation could be used for rehabilitative purposes as a means of enhancing activation of cortical compensatory reserves, counteracting potential negative interference and promoting the integration of neuronal circuits serving different functions.

## Introduction

Gait disorders are very common among elderly people and people with neurological disorders, causing 17% of all reported falls (Rubenstein, 2006; Axer et al., 2010). The cognitive contribution to gait has been recognized as a cause of postural instability and risk of falling in people showing normal aging and in people with neurodegenerative diseases. This has led to an increase in research into neural activation during walking, using a variety of techniques including near infrared spectroscopy (NIRS), positron emission tomography (PET), and electroencephalography (EEG) (Hamacher et al., 2015). Evaluation of how motor performance is affected by an additional cognitive load has also been used to assess risk of falling in healthy young and old subjects and patients with motor and cognitive impairments (Al-Yahya et al., 2011). The presence of cognitive engagement during gait refers to performance in multiple tasks, with temporal overlap of simultaneous executive functions. It is well-documented that impairment in dual-task (motor-cognitive) performance characterizes neurodegenerative diseases, including Parkinson's disease (PD) (O'Shea et al., 2002), Alzheimer's disease (Camicioli et al., 1997), and multiple sclerosis (Wajda et al., 2013). Although the functions of the musculoskeletal and biomechanical systems during gait are well-known (Kirtley, 2006; Perry and Burnfield, 2010), an understanding of the neuronal processes underlying stable gait is still lacking (Segev-Jacubovski et al., 2011). The dearth of data on cognitive processes during walking is probably due to the difficulty of assessing neuronal functions during the course of the movement. Changes to the spectral components of EEG rhythms in the alpha and beta ranges reflect activation of the motor system during walking (Jasper and Penfield, 1949; Pfurtscheller and Berghold, 1989; Pfurtscheller and Lopes da Silva, 1999; Androulidakis et al., 2007; Zhang et al., 2008; Pogosyan et al., 2009; Joundi et al., 2012; Solis-Escalante et al., 2012; Hamacher et al., 2015), which need to be assessed to understand the interference induced by cognitive engagement. However, EEG signals are susceptible to physiological and non-physiological artifacts, including motion artifacts, that can compromise the decoding of gait and the separation of neural signals related to bipedal locomotion. The EEG activity related to walking may be separated from physiological and non-physiological artifacts, including motion artifacts, using automatic recognition methods (Nathan and Contreras-Vidal, 2016).

In addition, the impact of cognitive interference on gait efficiency can be investigated by recording brain activity related to the cognitive task, as described in previous studies (De Sanctis et al., 2014).

Huntington's Disease (HD) is an autosomal dominant illness characterized by motor and cognitive impairments and psychiatric disturbances. The HD motor impairment is complex and includes akinesia, bradykinesia, and a progressive loss of coordination that affects functional ability (Van Vugt et al., 2004). A general impairment in motor planning cause difficulties of executive functioning, which may better emerge during multitasking experimental paradigms. Moreover, individuals with HD commonly experience falls (Busse et al., 2009), which may partly be the consequence of the inability to perform multiple executing functions, as walking during a contemporary cognitive task.

Recent studies showed that gait speed during a motor-cognitive dual task (walking whilst counting backwards) was correlated with United Huntington's Disease Rating Scale Total Motor Score (UHDRSM) (Huntington Study Group, 1996) and performance on a cognitive test (Delval et al., 2008; Fritz et al., 2016). As HD is a complex disorder in which the cognitive and motor symptoms are inter-related, we aimed to compare reciprocal interference between walking and a simple P3 odd-ball acoustic paradigm. Considering that the complexity of cortical engagement in walking activity may be described with the modification of EEG spectral components in alpha and beta ranges, the study aimed to evaluate the EEG and EMG correlates of this multitasking procedure in HD patients compared to age and sex matched controls. The study specifically intended to assess if (1) the P3 features would be modified differently by walking in HD patients and controls. (2) the changes of motor activity during walking due to cognitive engagement differed between HD patients and controls (3) the P3 task would induce different effects on EEG activities related to walking in HD patients and controls.

## Methods

The study was approved by Bari Policlinico General Hospital Ethical Committee, and all subjects provided written, informed consent to participation and publication of data that could identify them under a code. Preliminary results from healthy subjects were presented at the 6th IEEE International Workshop on Advances in Sensors and Interfaces, IWASI 2015 (de Tommaso et al., 2015). The work was carried out in accordance with the Code of Ethics of the World Medical Association (Declaration of Helsinki) for experiments involving humans (World Medical Association, 2013).

### Subjects

Twenty-four HD patients being followed at Apulian regional referral center for HD were enrolled in the study. Fourteen age- and sex-matched healthy volunteers were also examined. Demographic and clinical data for the patient group are reported in Table 1. Exclusion criteria were evidence of general medical or other neurological and psychiatric diseases and any kind of auditory impairment. Decisions about whether the exclusion criteria were met were based on a detailed interview, medical history, and objective general and neurological examinations. Patients who were able to walk along a short course without support were recruited. Two patients were excluded as they showed a marked tendency to fall and total incapacity to walk unaided during the task.

**Table 1:** Demographic and clinical characteristics of HD patients and controls.

**Patients**	**CAG**	**Duration (years)**	**Age**	**Sex**	**UHDRSM**	**Total chorea**	**Chorea lower limbs**	**Bradikinesia**	**Total dystonia**	**Dystonia lower limb**	**Walking**	**Tandem walking**	**Posture**	**Mini mental**	**Drugs**
1	51	8	35	M	88	17	4	4	3	0	4	4	4	27	Neuroleptics, SSRI
2	47	0.5	32	F	2	2	0	0	0	0	0	0	0	30	None
3	41	13	67	M	25	7	2	1	0	0	1	1	1	27	Neuroleptics, SSRI
4	53	2	29	F	15	5	2	0	1	0	0	1	1	27	None
5	42	1.7	45	M	16	5	2	0	1	0	0	0	0	25	Atypical neuroleptics
6	41	5	64	M	55	7	2	4	3	0	2	3	1	27	Neuroleptics, SSRI
7	45	1	42	F	36	7	2	2	1	0	1	2	0	23	None
8	41	26	50	F	44	8	2	2	2	0	2	2	1	18	Neuroleptics, SSRI
9	41	8	57	M	58	14	4	3	3	0	2	2	2	27	Neuroleptics, SSRI
10	46	6	53	M	51	9	2	2	8	2	2	2	1	26	Neuroleptics, SSRI
11	42	17	69	M	55	7	2	3	1	0	3	3	2	20	Neuroleptics, SSRI
12	49	5	38	F	49	4	1	2	9	6	2	4	1	19	Neuroleptics, SSRI
13	44	5	47	F	44	10	4	1	2	0	1	1	1	23	Neuroleptics, SSRI
14	42	6	52	M	48	7	2	2	4	1	1	2	2	25	Neuroleptics, SSRI
15	45	1	42	F	35	7	2	2	1	0	1	2	0	25	None
16	45	2	41	F	15	5	2	0	1	0	0	1	1	30	None
17	41	8.6	54	F	29	7	2	1	4	3	1	1	2	22	Neuroleptics, SSRI
18	48	8	35	M	42	6	2	2	6	2	1	1	1	28	Neuroleptics, SSRI
19	40	11	56	F	29	8	2	1	1	0	1	1	2	27	Neuroleptics, SSRI, tricyclic antidepressants
20	47	0.7	30	M	3	0	0	0	0	0	0	0	0	23	None
21	47	7	43	M	47	12	4	2	1	0	2	3	1	23	Neuroleptics, SSRI
22	42	5	59	F	65	13	4	2	9	4	1	2	1	23	Neuroleptics, SSRI
23	40	5.7	67	F	25	3	2	1	4	0	1	1	1	23	None
24	44	8	59	M	30	8	2	2	4	1	1	2	1	29	Neuroleptics, SSRI
HD patients			M = 48.13, *SD* = 11.53	12 M, 12 F	M = 37.71, SD = 20.21	M = 7.4, *SD* = 3.8	M = 2.21, *SD* = 1.12	M = 1.6, *SD* = 1.1	M = 2.8, *SD* = 2.7	M = 0.79, *SD* = 1.5	M = 1.25, *SD* = 0.98	M = 1.7, *SD* = 1.12	M = 1.12, *SD* = 1.89	M = 24.87, *SD* = 3.24	
Controls			*M* = 48.8, *SD* = 14.13, ANOVA: *F* = 1.23, n.s.	7 M, 7 F, χ^2^ = 1.21, n.s.											

*Total Unified Huntington's Disease Motor Rating Scale (UHDRSM) score and some item scores are reported. The tandem walking is a gait where the toes of the back foot touch the heel of the front foot at each step. SSRI: selective serotonin reuptake inhibitor*.

Patients underwent the motor section of Unified Huntington's Disease Rating Scales, (UHDRS) (Huntington Study Group, 1996) and the Mini Mental State Examination (Folstein et al., 1975). The distribution of age and sex was similar in the patient and control groups (Table 1).

### Recording

EEG—EMG recordings were obtained using MICROMED EEG apparatus Micromed Brain Quick, Mogliano Veneto, Italy). EEGs were recorded using a prewired head cap with 21 Ag-AgCl surface electrodes; further electrodes above the right and left eyebrows that were referenced to the nasion were used for the electro-oculogram (EOG). A 0.1–100 Hz band-pass filter with a 50 Hz digital filter was applied during recording of EEG data. Further derivations were used to record electromyography (EMG) signals from the right and left anterior tibialis and right and left lateral gastrocnemius, using superficial Ag-AgCl electrodes fixed by collodion. The ground electrode was positioned over the cervical zone. The amplifier box was carried in a backpack and electrode cables were carefully fixed to the legs. All subjects wore a wrist pedometer.

### P3 task

The auditory task was controlled via the Brain Quick Micromed program. Two types of acoustic stimuli (50 rare stimuli; 150 frequent stimuli) were delivered at random intervals (1–3 s). The stimuli consisted of pure tones of 70 dBL SPL intensity with a duration of 100 ms and a rise and fall time of 10 ms. The frequency of the rare stimulus was 1,000 Hz and the frequency of the frequent stimulus was 250 Hz. The sounds were delivered freely in the experimental environment using two loudspeakers; subjects did not wear acoustic cups and were asked to attend to and count the rare stimuli. The output variable was total number of errors (omissions and false hits).

### Experimental procedure

The experiment was carried out in a quiet and soundless room in which a 10 m by 5 m path had been marked out. Subjects were asked to walk up and down this path, walking as naturally as possible, whilst wearing the electrodes and carrying the backpack on their shoulders. Data were recorded whilst subjects were standing for 5 min (C1); walking for 5 min (C2); standing for 5 min whilst performing the P3 odd-ball task (C3); walking for 5 min whilst performing the P3 odd-ball task (C4). The sequence of the four experimental conditions was randomized across patients and controls. All the subjects were tested during the morning. Walking speed was evaluated through the pedometer.

### EEG-EMG analysis

The ASA software, vers. 4.7.3.1 by ANT software (http://www.ant-neuro.com/products/asa) was used for EEG and EMG analysis. Both EEG and EMG signals were sampled at 256 Hz. A multi-step artifact removal procedure was applied to ongoing EEG in order to generate reliable EEG signals and event-related potentials for analysis. After visual inspection of EEG recordings, the frequency, and amplitude of electrode oscillations were characterized on a per subject basis. An automatic artifact recognition and removing was previously performed for the slow oscillations present on the EOG and EMG channels, exceeding 150 μV amplitude. The remaining EEG recording was corrected using a principal component analysis (PCA) method that models the brain signal and artifact subspaces (Ille et al., 2002), according to ASA software. PCA is a reliable method of extracting EEG components based on temporal and spatial features. This method separates brain signals from artifacts, removing the artifacts without significantly distorting the EEG. It is well-applied to extract event-related potentials (Dien et al., 2007), and may also support the recognition and consequent removal of well-defined artifact activities. (ter Braack et al., 2013). The artifact correction method, implemented in ASA software, uses two criteria to determine which part of the data is considered signal (data subspace). The first criterion specifies the highest permitted amplitude of the brain signal and the second criterion specifies the highest permitted correlation between brain signal and artifact topography. PCA is then used to determine the topographies of the artifact-free signals and the artifacts. Separation was achieved by means of data intervals with a clear artifact activity. We implemented PCA separately for the different types of artifact, which were marked for the following analysis: (1) repetitive electrode oscillations during walking (activity in the 0.5–1 Hz range present on the 21 scalp electrodes; (2) eye movements in the 0.5–2 Hz frequency range, with prevalent amplitude over the frontal (Fp1,Fpz,Fp2) electrodes; (3) rarer low frequency, non-repetitive electrode oscillations in HD patients due to choreic movements, which persisted after automatic rejection of activity exceeding 150 μV. The time basis considered was the longest duration we have marked, the amplitude threshold was 100 μV, the maximal correlation with artifact subspace was settled at the default value of 50%, the minimal variance with the data subspace at the default value of 10%. A similar PCA was applied to EMG recordings to subtract the main slow artifacts. We took into consideration the limited reliability of dynamic spectral analysis (Farina, 2006) and simply considered the total spectral power of EMG activity in the walking and walking+P3 conditions, filtered in the 10–90 Hz frequency range and normalized by subtracting standing condition values on a per subject basis.

The modifications of spectral components of ongoing EEG activity, were evaluated by filtering the EEG in the 7–12 and 13–30 Hz frequency ranges (corresponding to alpha-mu and beta rhythms). In fact these frequencies are specifically modified during walking, and could describe the interference due to a contemporary cognitive task (Hamacher et al., 2015).

For the cognitive task, we averaged at least 30 artifact-free EEG recordings corresponding to presentation of rare and frequent stimuli and extracted the P3 component, considering 100 ms as prestimulus and 900 ms as poststimulus times with a baseline-correction for dc (direct current) offset subtraction with 0.5 s duration, according to ASA software Version 4.3.1. For P3 analysis, the EEG was filtered in the 0.1–70 Hz frequency range. We performed a semiautomatic peak detection with the maximum area of the P3 wave, which considered at least 50% amplitude prevalence of the positive wave in the time range 200–500 ms obtained by the rare and frequent stimuli. The results of semiautomatic analysis were validated by visual inspection of the data.

### Statistical analysis

We assessed the normality of the distribution of data using the Kruskal-Wallis test. A preliminary MANOVA analysis with EEG channels as variables and condition rare-frequent stimuli as factors, was employed separately in HD and control groups to ensure the reliability of P3 wave. P3 wave amplitude, P3 latency and log-transformed EEG and EMG spectral values were evaluated by multivariate analysis of variance (MANOVA) (complete factorial model, sum of squares type III) with EEG and EMG channels as variables. The main MANOVA factors were condition (for EEG bands: standing for 5 min -C1- vs. walking for 5 min -C2- vs. standing for 5 min whilst performing the P3 odd-ball task -C3- vs. walking for 5 min whilst performing the P3 odd-ball task -C4-; for P3 amplitude: standing for 5 min whilst performing the P3 odd-ball task -C3- vs. walking for 5 min whilst performing the P3 odd ball task; for normalized EMG spectral components: walking for 5 min -C2- vs. walking for 5 min whilst performing the P3 odd-ball task -C4-) and group (HD vs. control). Considering that age had a consistent variability within groups, we included it in the MANOVA as a control variable. Separate *post-hoc* Bonferroni tests (for EEG bands comparing C1 vs. C2 vs. C3 vs. C4) and paired-sample *t-*tests with Bonferroni correction (for P3 amplitude, P3 latency, C3 vs. C4, for normalized EMG spectral component; C2 vs. C4) were carried out for each group using SPSS v. 21.

We also employed a linear regression test with the main clinical features of HD as independent factors and P3 amplitude and EMG spectral components as dependent variables, using the condition walking vs. standing (C3-C4) and walking without and during the P3 (C2 and C4), as selection variables.

The main P3 topography was represented using Scalp Maps, provided by ASA software (4.7.3 software version, by ANT software http://www.ant-neuro.com/products/asa). The detailed analysis, including spectral data and amplitudes, as well as the detailed statistical analysis is presented in a supplementary section.

## Results

### Task performance

All subjects performed the walking task. The control group's mean walking speed was 1.9 m/s ± 0.23 in the single-task condition C2 and 1.88 m/s ± 0.24 in the dual-task condition C4 (walking+P3 task). The patient group's mean walking speed was 1.21 m/s ± 0.44 in the single-task condition C2 and 1.19 m/s ± 0.38 in the dual-task condition C4. ANOVA showed an effect of diagnosis (*F* = 7.37, *p* < 0.01), but there was no effect of condition (*F* = 1.23, n.s.) and no condition x diagnosis interaction (*F* = 1.45, n.s.). For the control group the target stimuli errors rate was 2.3 ± 0.5 in the single-task condition C3 and 2.4 ±0.8 in the dual-task condition C4 (walking+P3). For the patient group it was 3.1 ± 0.9 in the single-task condition C3 and 3.2 ± 0.8 in the dual-task condition C4. ANOVA indicated that there was no effect of diagnosis or condition on performance of the P3 task.

### P3 amplitude

**Figure 1:**
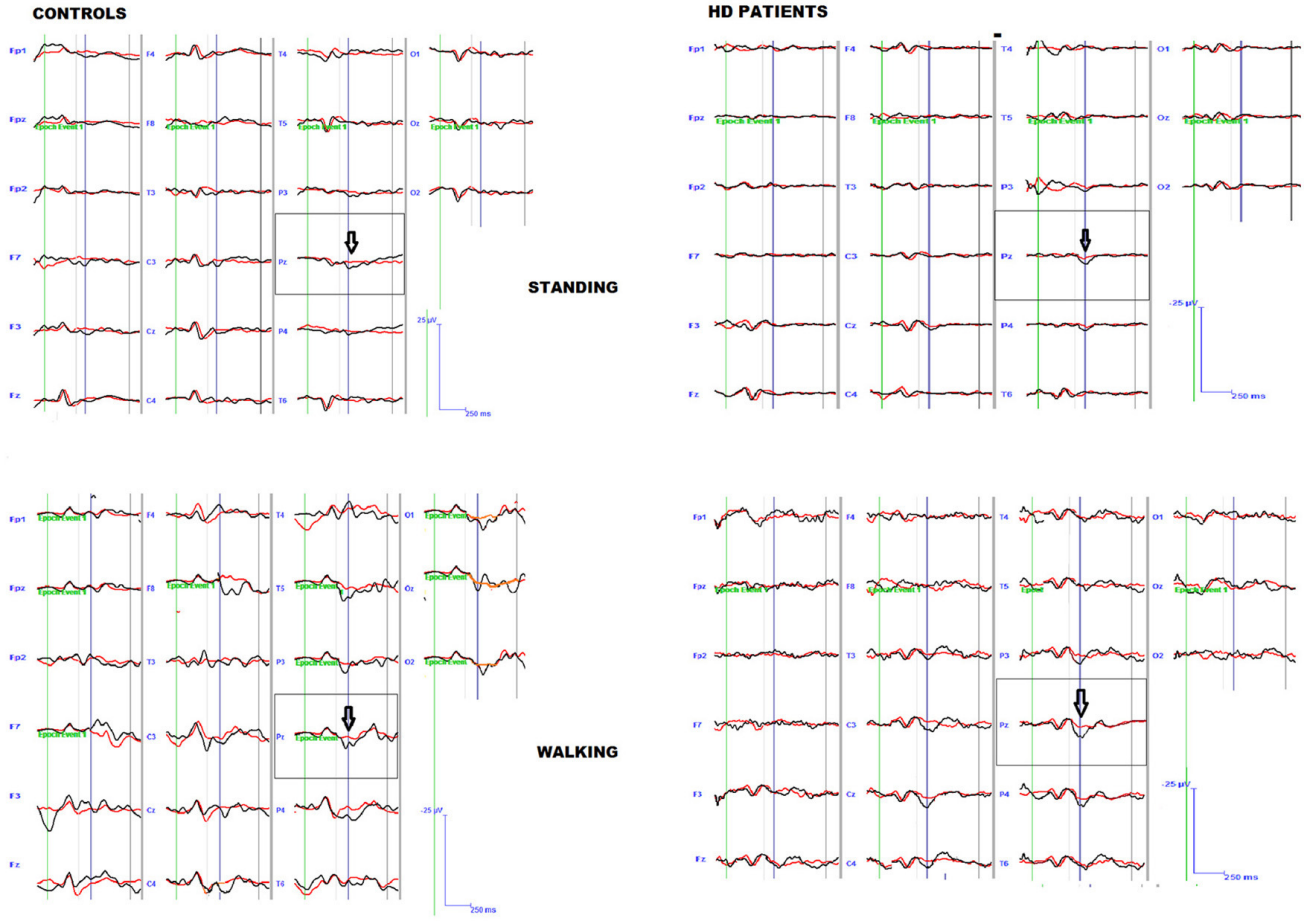
Grand average of the P3 wave by target (black line) and non-target (red line) stimuli in patients and controls, during standing and walking conditions.

**Figure 2:**
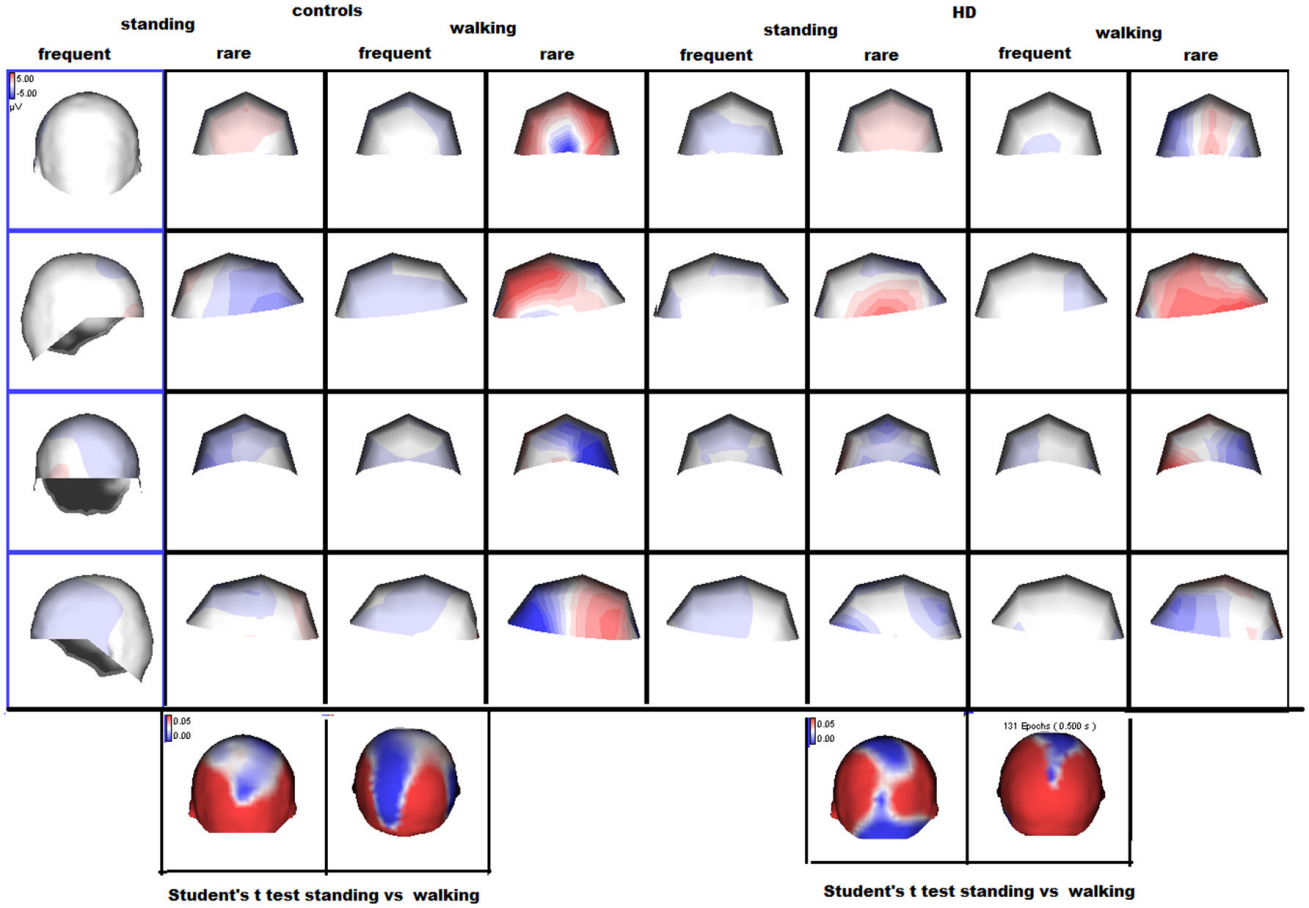
Scalp maps of the P3 wave by rare and frequent stimuli are reported. The amplitude maps at the bottom of the figure represent the results of separate Student's *t*-tests for each group, where the color scale indicates the *p*-values. Significant results are shown in white and blue.

The preliminary MANOVA analysis assessed a significant amplitude prevalence of the response in the 200–500 ms time interval to the rare stimulus compared to the frequent one (controls *F*-value-Roy's largest root- 2.99 hypothesis DF 21, DF 1; error DF 6 *p* < 0.01; HD F 2.48, hypothesis DF 21, DF 1, error DF 26 *p* < 0.01 in standing condition; controls F 3.01 *p* < 0.01; HD 2.55 *p* < 0.01 in walking condition). The P3 amplitude was similar in both groups, and condition (walking C4 vs. standing C3) had a significant effect in both groups (Table 2, Table S1), though this was more evident in controls (Figures 1, 2, Tables S1, S2).

**Table 2:** MANOVA analysis (Roy's largest root) for alpha and beta rhythm, muscular activity, P3 amplitude, and latency with electrodes (21 for alpha and beta activity and P3 amplitude, 4 for EMG activity) as variables and diagnosis controls vs. HD and conditions walking vs standing vs. P3 walking vs. P3 standing as factors.

	**F**	**Hypothesis DF**	**DF**	**Error DF**	**Sig**.
**P300 AMPLITUDE**					
Diagnosis	0.0714	21	1	52	*n*.*s*
Condition	2.5	21	1	52	0.01
Diagnosis × Condition	2.1	21	1	52	0.04
**ALPHA RHYTHM**					
Diagnosis	4.29	21	1	292	0.0001
Condition	4.97	21	3	290	0.0001
Diagnosis × Condition	2.29	21	3	290	0.008
**BETA RHYTHM**					
Diagnosis	3.38	21	1	292	0.0001
Condition	3.83	21	3	290	0.0001
Diagnosis × Condition	1.72	21	3	290	0.042
**10–90 Hz MUSCLE ACTIVITY (NORMALIZED)**					
Diagnosis	9.75	4	1	79	0.0001
Condition	1.33	4	1	79	*n*.*s*.
Diagnosis × Condition	0.73	4	1	79	*n*.*s*.

### P3 latency

**Figure 3:**
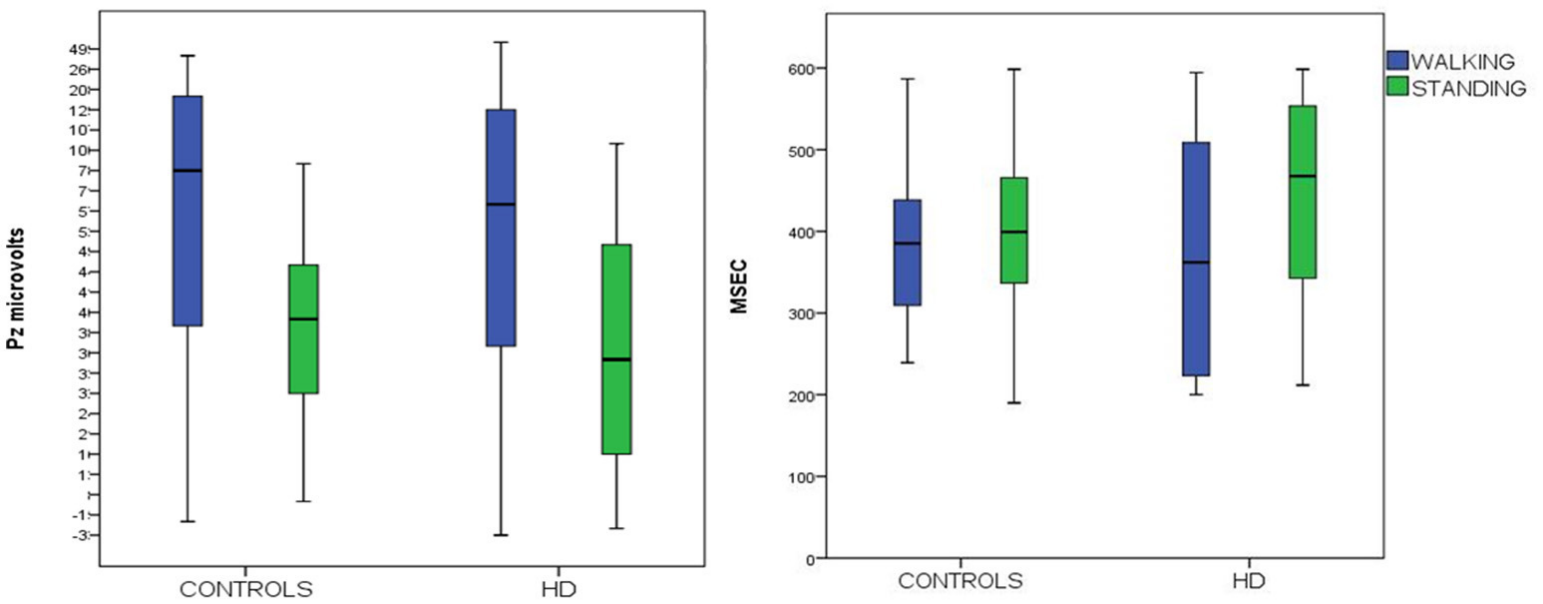
Means and 95% confidence intervals for P3 amplitude (on the right) and latency (on the left) to target stimuli, recorded over the Pz channels in HD patients and controls, during standing and walking conditions.

P3 latency measured at the Pz channel was similar in the two groups. Both groups showed a non-significant decrease in walking conditions C4 (Figure 3, Table 3).

**Table 3:** ANOVA analysis.

**P3 latency**	**F**	**DF**	**Sig**.	**Error**
Diagnosis	2.52	1	n.s	
Condition	0.55	1	n.s.	
Diagnosis × Condition	0.44′	1	n.s.	
				72

*P3 latency computed over the Pz electrode as factor*.

### EMG/EEG activity power spectra

**Figure 4:**
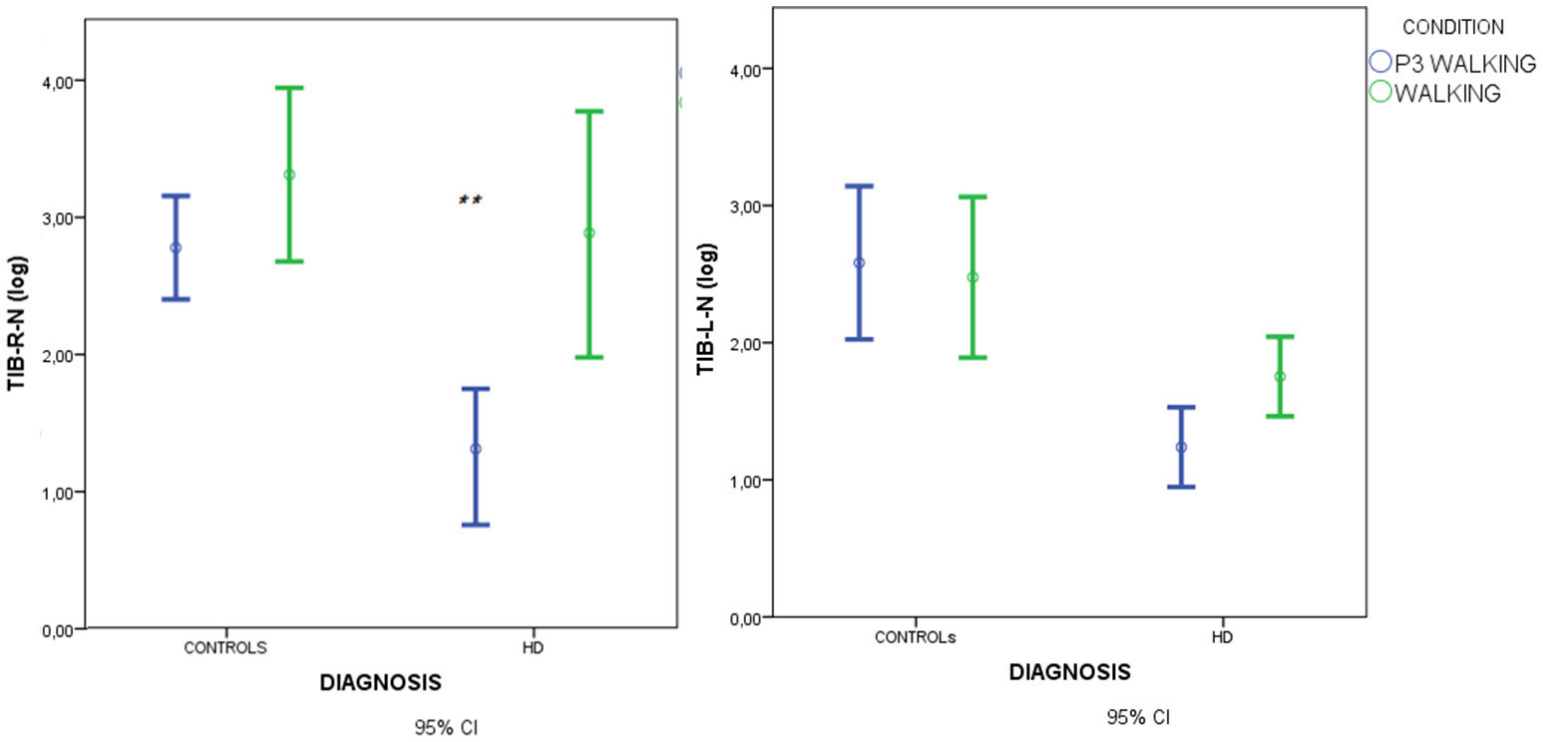
Means and 95% confidence intervals (CI) for the total spectral power of EMG activity, normalized by standing condition values and log-transformed, in the walking and walking+P3 conditions in HD patients and controls. Values from right anterior tibial (TIB-R-N-log) and left anterior tibial (TIB-L-N-log) muscles are reported. Results of separate paired-samples Student's *t*-tests for each group: ^**^p < 0.01.

**Figure 5:**
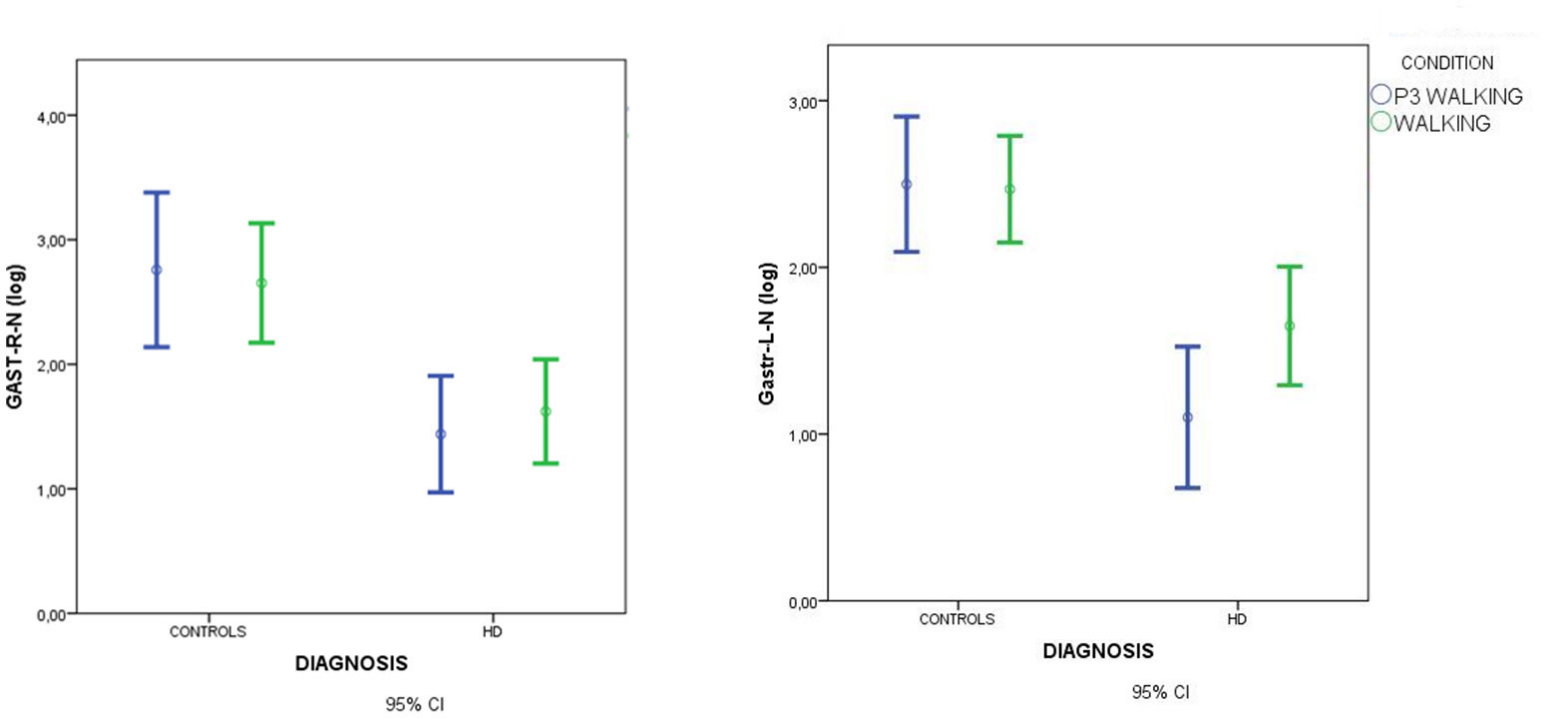
Means and 95% confidence intervals (CI) for total spectral power of EMG activity, normalized by standing condition values and log-transformed, in the walking and walking+P3 conditions in HD patients and controls. Values from right gastrocnemius (GSTR-R-N-log) and left gastrocnemius (GASTR-L-N-log) muscles are reported. Separate paired-samples Student's *t*-tests for the two groups were not significant.

Spectral analysis of muscle activity during walking C2 and walking+P3 conditions C4 was standardized by subtracting standing condition activity on a per subject basis. There was a main effect of diagnosis but no effect of condition and no diagnosis × condition interaction (Figures 4, 5, Table 3).

**Figure 6:**
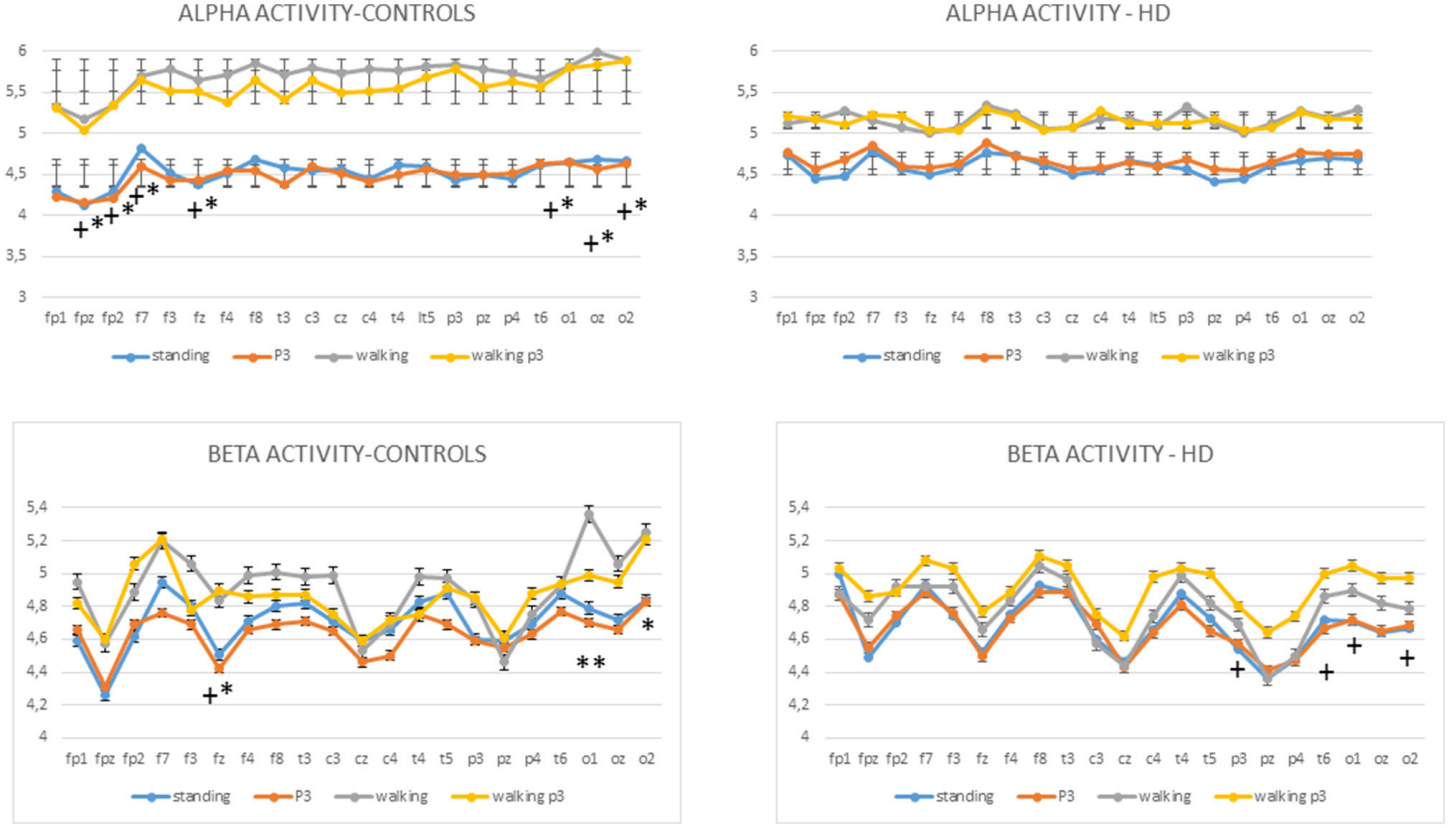
Mean values and standard errors for alpha activity (top) and beta activity (bottom) in controls and HD patients. The results of separate Bonferroni tests for each group are reported, standing vs. walking and standing+P3 vs. walking+P3: ^*^p < 0.05, ^**^p < 0.01; standing vs. walking+P3 and standing+P3 vs. walking+P3: ^+^*p* < 0.05.

In HD patients descriptive data suggested that muscle recruitment during walking was further reduced during the dual-task condition (P3+walking, C4) (Figures 5, 6), but Student's *t*-tests showed that the reduction was only significant for the right anterior tibial muscle. (Figures 4, 5).

#### Alpha activity

The MANOVA model showed that there were main effects of diagnosis and condition on alpha activity as well as a diagnosis × condition interaction (Table 2). The cognitive task did not cause a change in alpha activity in the standing C3 or walking conditions C4. In controls alpha activity was greater in the walking C2 and P3+walking conditions C4 compared to the standing C1 and P3+standing C3 conditions respectively, over several electrodes, in particular the frontal and temporo-occipital electrodes (Figure 6, Table 2, Table S3). In HD patients the alpha activity in standing C1 and P3 standing C3 conditions were quite similar to normal values, while in the course of walking and P3 walking (C2, C4) there was only a slight and not significant increase of alpha power. (Figure 6).

#### Beta activity

MANOVA also showed effects of diagnosis and condition on beta activity, as well as a diagnosis × condition interaction (Figure 5, Table 2, Table S4,). In both HD patients and controls, the beta power did not change significantly in the standing C1 vs. P3 standing C3 and walking C2 vs. P3 walking C4 conditions (Figure 6). In controls the Bonferroni test showed a significant increase of beta power in the walking C2 and P3 walking C4 vs. standing C1 and P3 standing C3 conditions interesting one frontal electrode and the bilateral occipital derivations (Figure 6). In HD patients, the beta rhythm was increased in walking P3 task C4 over the temporo-parietal electrodes as compared to the standing C1 and P3 standing C3 conditions. (Figure 6, Table S4).

### Linear regression analysis

**Figure 7:**
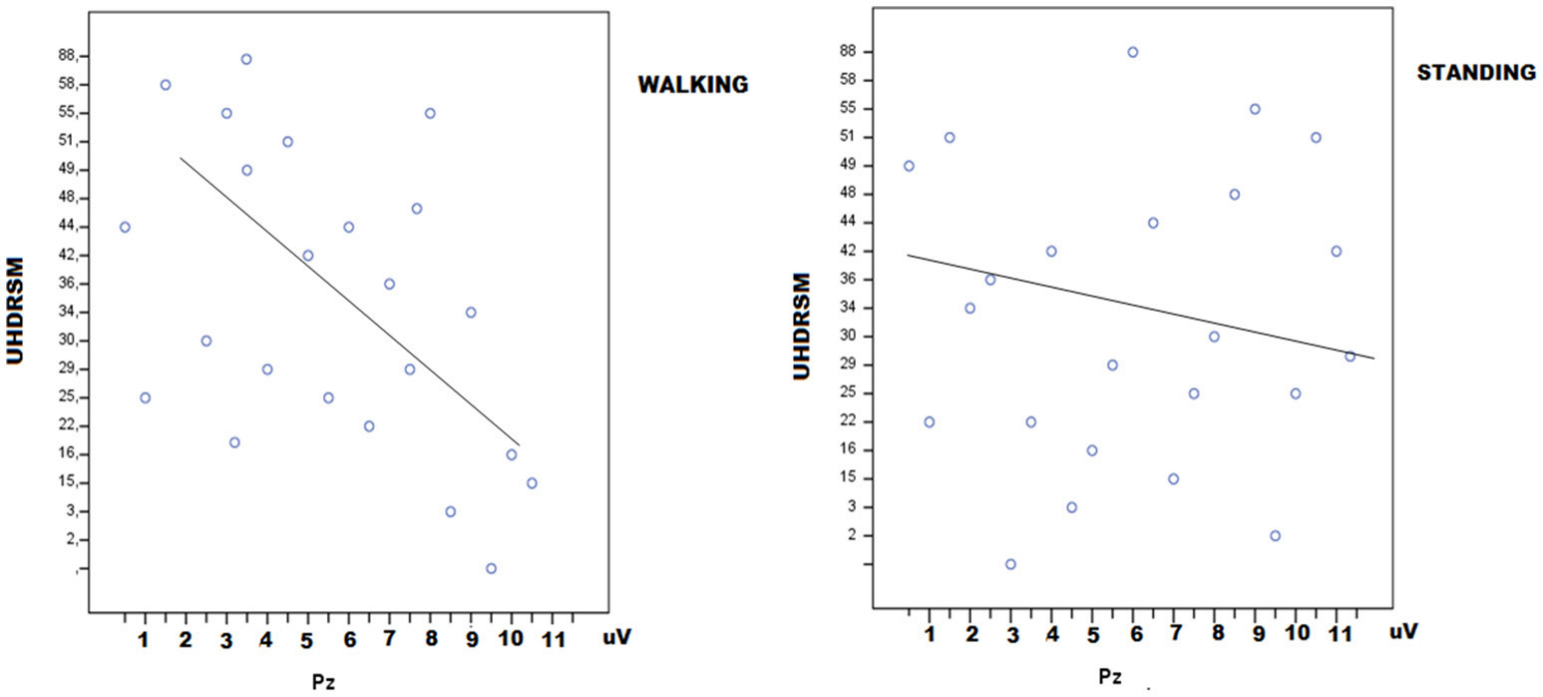
Linear dispersion graph for UHDRSM scores (Unified Huntington's Disease Rating Scale-Motor) and P3 amplitude over the Pz electrode in walking and standing conditions. A negative correlation was present in the walking condition (detailed statistical results are reported in Table 4).

**Figure 8:**
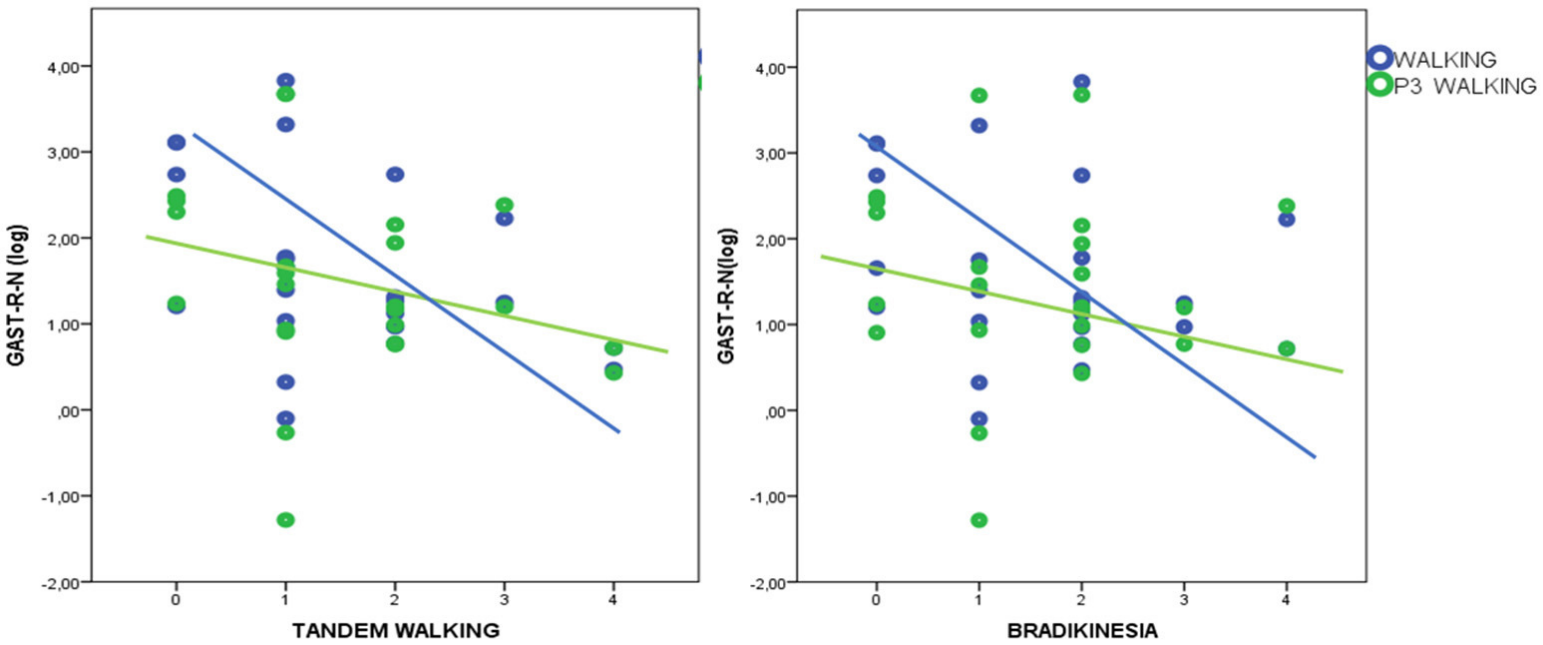
Linear dispersion graph for bradykinesia and tandem walking (Unified Huntington's Disease Rating Scale-Motor items) and right gastrocnemius muscle recruitment (total spectral power of EMG activity, normalized by standing condition values and log-transformed) during walking and walking+P3. A negative correlation was present only in the walking condition (detailed statistical results are reported in Table S5).

In HD patients P3 amplitude was negatively correlated with UHDRSM, bradykinesia, and walking scores in the walking C4 condition, but not in the standing C3 condition (Figure 7, Table 4). During the walking task C2 recruitment of the right gastrocnemius and anterior tibial muscles was negatively correlated with illness duration (Table S5). The muscle recruitment of the right gastrocnemius and anterior tibial muscles during the walking task C2, was inversely correlated with illness duration, the muscle recruitment of the right gastrocnemius was also negatively correlated with bradykinesia, walking, and tandem walking items. (Table S5). This correlation was absent when patients performed the P3 task in the C4 condition(Figure 8, Table S5).

**Table 4:** Linear regression analysis for P3 amplitude on Pz electrode and main clinical features in HD patients UHDRSM.

	**DF**	**F (walking)**	**Sig**	**F (standing)**	**Sig**
Linear regression analysis	12	3.648	0.024	1.067	0.466
**Single independent variables**		***t* (Walking)**		***t* (Standing)**	**Sig**.
CAG		1.006	0.338	1.252	0.239
Duration		1.313	0.218	1.715	0.117
UHDRSM		3.442	**0.006**	0.305	0.767
Chorea		1.799	0.102	0.966	0.357
Chorea lower limb		2.145	0.058	0.331	0.748
Bradikinesia		2.737	**0.021**	0.116	0.910
Dystonia		1.379	0.198	−1.112	0.292
Diystonia lower limb		1.463	0.174	1.580	0.145
Walking		2.963	**0.014**	0.290	0.778
Tandem Walking		1.979	0.076	0.445	0.666
Posture		1.231	0.246	1.292	0.225
MMSE		2.121	0.060	−1.415	0.187

*Motor section of Unified Huntington's Disease Rating Scale. Statistical significance are outlined in bold*.

ANOVA indicated that in the patient group neuroleptic treatment did not affect any of the outcome variables—P3 amplitude, P3 latency, alpha, and beta activity and muscle recruitment.

## Discussion

The results of this study could indicate that the P3 features were not substantially dissimilar between patients and controls, and that its amplitude appeared enlarged during walking in both groups, though this phenomenon was less evident in patients. The cognitive engagement did not cause a deterioration of motor performance in controls and patients, though in the latter group it was associated with a slight reduction of muscle recruitment. The EEG spectral correlates of walking in the alpha and beta frequency ranges, were generally increased during movement in control subjects and not significantly modified by concurrent P3 task. In HD patients this effect was evident in regard to the beta rhythm.

The discussion is organized as follows: comments to the main results, followed by the limitations of the study and general conclusions.

### P3 features in basal conditions and walking conditions

P3 amplitude increased during walking in both HD patients and controls. In the basal conditions there were no group differences in P3 amplitude or latency. All the patients included in this study were capable of independent walking and had normal or slightly reduced MMSE scores, i.e., had mild symptoms of HD. The nature and extent of P3 abnormalities vary between HD cohorts. In an earlier study of early-stage HD patients and at-risk presymptomatic subjects we found that the P3 latencies of the majority of HD patients and all presymptomatic gene carriers were within the normal range (de Tommaso et al., 2003b). More recent studies have shown that the latency of the P3 wave is increased at several stages of HD (Beste et al., 2010; Hart et al., 2012, 2015). These apparent discrepancies may be due to differences in the experimental procedures used in the P3 task and the different clinical conditions of the patient samples. In our procedure, subjects were required to detect an acoustic target but did not use a motor action to indicate target detection, while most of the studies which have found abnormal P3 latencies have used go/no-go paradigms, that could reveal deficits in pre-motor inhibition and motor preparation (Beste et al., 2010; Hart et al., 2012, 2015). A clear increase in P3 latency was observed in a visual task, with a delay affecting the early visual components; this result suggests that HD patients may have a particular problem with visual stimulus processing (Muente et al., 1997). Compensatory mechanisms for coping with specific cognitive deficits and paradoxical enhancement of cognitive functions not specifically related to motor performances have also been described in HD (Beste et al., 2014; Hart et al., 2015). Our failure to find P3 abnormalities in our HD series may be due to our use of a purely auditory task that does not require a motor response and our recruitment of patients with only slight or moderate cognitive impairment. The percent rate of errors in counting the target stimuli was non-significantly higher in HD patients, confirming that the cognitive process of stimulus recognition is normal in the early and middle stages of the disease when motor preparation for a go or no-go response is not required.

Walking produced a clear increase in P3 amplitude in both groups. This is a novel finding as few studies have evaluated event-related brain activity with dual-task paradigms. In P3 studies on healthy volunteers comparing sitting in a quiet room with walking in environments with substantial ambient noise, the response to target stimuli seemed reduced during outdoor movement, an effect partly attributable to the environmental distractors as busy streets and traffic (Debener et al., 2012). A previous study that evaluated the effect of treadmill walking on the performance of a visual P3 go/no-go task in healthy subjects, found a reduction in the amplitude of the N2-P3 inter-peak amplitude and an increase in its latency in the no-go target condition (De Sanctis et al., 2014). In that study the P3 task involved a motor response and hence potential recalibration of the cortical resources engaged in the inhibitory motor task to optimize performance in dual-task contexts (De Sanctis et al., 2014). The same authors also found an increase in amplitude of the P3 peak over the central sites, indicating that even in this dual-task context the P3-related processing was improved when subjects were walking.

Our task was different as it was based on acoustic discrimination of the rare stimulus, with no involvement of cortical resources competing with the walking task. With our task the act of walking seemed to increase rather than reduce cortical involvement in the acoustic discrimination task, even in HD patients. The P3 showed a typical amplitude distribution over the central-parietal regions across the midline in both groups, although the increase was more topographically restricted in HD patients. Previous dual-task studies have reported that the majority of HD subjects experienced interference between gait and cognitive performance, such that under dual-task conditions cognitive performance decreased when gait speed increased (Fritz et al., 2016). Accordingly, in people with neurologic disease, the attention demanding exercise of walking affect the cognitive resources (Yogev-Seligmann et al., 2008), requiring greater cortical activation. In the present study we employed a standard P3 auditory task to record reliable event related responses, avoiding to use the typical alphabetic paradigm (Fritz et al., 2016). This type of cognitive test did not change walking speed in controls during the P3 task and only slightly reduced it in HD patients, suggesting that the interference with walking is related to the type of cognitive engagement. However, in HD patients the P3 task caused a reduction in muscle recruitment, suggesting a slight influence on motor performance. Dynamic exercise can improve cognitive function and increase blood flow within the prefrontal cortex (Endo et al., 2013). Extensive cortical activation including the supplementary motor area (SMA), frontal gyrus, insula, and cingulate cortex has been observed in walking (Hamacher et al., 2015). The SMA plays important, albeit different, roles in various cognitive domains including action, temporal, and spatial processing, numerical cognition, music, and language processing and working memory (Cona and Semenza, 2016). The increase in P3 amplitude observed during walking in both healthy subjects and HD patients suggests that the cortical regions that generate this wave were activated rather than inhibited by the contemporary movement. However, the increased P3 amplitude was not associated with a real cognitive facilitation, i.e., reduced detection latency and better recognition performance, instead it was accompanied by preservation of single-task levels of P3 performance during the dual-task condition. Activity in cortical regions responsible for integrating use of motor and cognitive executive functions may have been responsible for preservation of cognitive ability during walking and this probably accounts for the more extensive cortical activation we observed under dual-task conditions. Further research is needed to determine the extent to which motor activity facilitates the execution of cognitive tasks in HD patients. The hypothesis that motor activity facilitates cognitive processing was derived from studies showing physical exercise retards neurodegeneration in Alzheimer's disease (Okonkwo et al., 2014). Another point worth of deep examination would be the type of cognitive engagement subjected to possible facilitation rather than inhibition during walking, maybe a pure cognitive task not requiring motor action. In HD patients, the motor impairment, measured as UHDRM score, was negatively correlated with P3 amplitude during walking. This may be due to sensory feedback to the cortical regions responsible for motor and cognitive strategies from the body parts involved in walking. Functional magnetic resonance imaging (FMRI) studies have revealed that in PD the motor impairment caused by freezing reduces patients' capacity to recruit specific cortical and subcortical regions within the cognitive control network (Shine et al., 2013). Similarly, HD patients with more severe motor impairment showed reduced activation of cortical regions subtending P3 scalp representation during walking relative to patients with a less severe motor impairment. Increased recruitment of the cortical regions generating the P3 component during walking seems to be dependent upon the motor efficiency of HD patients.

### EMG power spectra and walking speed

The P3 acoustic task did not reduce the ability of motor task execution and walking speed in controls or HD patients, unlike the alphabetic test usually used in dual-task paradigms (Verghese et al., 2002). This suggests that the type of cognitive processing required to perform the acoustic odd-ball task does not have a negative effect on walking performance. Studies in PD patients have shown that rhythmic auditory stimulation improves motor functions and balance (Song et al., 2015). Our P3 task involved random acoustic stimulation that may have enhanced the rhythmicity of walking without competing with walking for attentional resources. Overall muscular recruitment during walking was reduced in HD patients, although the P3 task only had a negative effect on muscle activation at one site, the tibial anterior muscle, with a similar trend in controls. The anterior tibial muscle has a primary role during walking (Montgomery et al., 2016) and this may explain why it was subjected to interference from concurrent performance of the cognitive task. In HD patients motor impairment, as reflected in bradykinesia and deficits in the UHDRSM walking items, was correlated with reduced muscular recruitment. However, this correlation was absent in the dual-task condition, presumably because the attentional demands of the cognitive task caused a further reduction of muscular recruitment. This finding suggests that the acoustic P3 task could in part cause a deterioration of motor performance during walking, also in patients with better motor abilities, although use of compensatory motor strategies may contribute to preserve motor speed.

### EEG spectral analysis- alpha and beta activity changes during walking

Unlike previous studies we did not find a reduction in alpha power in our HD patients in the basal condition but we observed a scarce alpha rhythm modulation in the walking conditions. In the control group there was a clear increase in alpha activity over the frontal and occipital regions in the walking and walking+P3 conditions, but this effect was much smaller in the HD group. Previous studies have reported that a closed-eyes condition reduces alpha power in HD patients (Scott et al., 1972; Bylsma et al., 1994; de Tommaso et al., 2003a; Bellotti et al., 2004; Painold et al., 2010). This finding, together with our results, suggests that in HD patients modulation of the amplitude and synchronization of EEG rhythms in the alpha band may be impaired in several conditions, including eyes closed and active movement. During walking our control group displayed a clear increase in alpha band EEG activity over the frontal regions that probably correlates with activation of the motor cortical network (Hamacher et al., 2015). In high density EEG studies an alternating synchronization-desynchronization pattern was observed during treadmill walking, accompanied by fluctuating alpha and beta amplitudes during the gait cycle (Gwin et al., 2011; Hamacher et al., 2015). The alternating synchronization-desynchronization of alpha and beta bands during a single step sequence, may be totally in favor of an increase in EEG power in these frequency ranges across the whole gait cycle (Severens et al., 2012), which would account for the results we obtained in controls when we computed the EEG spectrum components over the entire walking task. In controls, there was a bilateral increase in alpha power over the frontal regions during walking, a finding which is compatible with the notion of cortical sources in prefrontal cortex, as indicated by high density EEG (Gwin et al., 2011). We also observed an increase in alpha power over the occipital electrodes, probably due to activation of the posterior regions involved in the visual exploration of the walking route, or to a diffusion of activity from the posterior parietal sources (Gwin et al., 2011). Severens et al. (2012), demonstrated that there was more beta and gamma band synchronization than alpha synchronization in EEGs recorded during walking, as a consequence of movement-induced artifacts, so our finding of a prevalent alpha band more than beta band increase during walking seems to be reliably attributable to walking-related cortical activation. Moreover, artifacts would be even more expressed in HD patients, who, despite this, showed a smaller increase in alpha band power during walking. In controls the trend in beta activity was similar to the changes in alpha activity, though this effect was limited to few frontal and posterior electrodes. The P3 task did not cause detectable modification of the alpha and beta power representation in either the standing or walking conditions, presumably because the P3 task is mainly associated with activity in the delta and theta bands (Basar-Eroglu et al., 1992; Yordanova and Kolev, 1998; Karakaş et al., 2000), while changes in alpha and beta bands are limited to the prestimulus time (De Blasio et al., 2013). Moreover, there is evidence that increased alpha power is generally associated with better cognitive performance, so the EEG activation induced by walking should facilitate cortical recruitment to the odd-ball task (Ramos-Loyo et al., 2004). In HD patients, the lack of increase in alpha activity during walking may be due to a basic defect in EEG rhythm modulation, rather than to cognitive interference. In HD patients the dual-task condition produced modulation of the beta rhythm over the temporal-parietal-occipital regions. Reports of abnormalities of beta rhythm in HD samples have been inconsistent (de Tommaso et al., 2003a; Painold et al., 2010), suggesting that beta rhythm is slight affected by the basal mechanisms of the disease, and prone to modulation by cognitive and motor tasks. In HD patients the increase in beta band power over the parietal and occipital electrodes appears to reflect visual perceptual processing during walking, which became evident during the P3 task. This suggests that in HD patients cortical compensatory mechanisms may be used to preserve cognitive performance during motor tasks.

## Study limitations

Despite we were able to detect spontaneous and evoked EEG activity even during dynamic condition in patients affected by movement disorders, the study suffers from main methodological flaws, as we employed standard EEG apparatus, which did not allow us to record accelerometer parameters or obtain reliable data on walking performance. In addition, the presence of cable connections made walking more difficult, especially for the HD patients.

Our primary aim was to evaluate the mutual interference between the EEG-EMG correlates of walking and the P3 wave, but the effect of cognitive interference on motor performance would be better investigated using wireless apparatus. Using a real walking route rather than a treadmill task increased the ecological validity of our walking task. Our artifact removal technique appeared to be efficacious for most of the EEG and EMG patterns we investigated, though we decided to limit our event-related potentials analysis to the P3 response to reduce the possibility of confounding results.

Finally, our HD group included patients taking neuroleptic medication. This subgroup appeared to have similar EEG characteristics to the untreated subgroup, but the possibility that there were subtle, drug-induced modifications of EEG rhythms cannot be excluded.

## Conclusions

The main significance of our findings is that in HD patients and controls the cortical activation during P3 task increased during walking, without adversely affect the changes of EEG alpha and beta bands induced by movement. This is in favor of a positive interference between walking and certain modalities of cognitive paradigms, as the general brain activation induced by walking may facilitate the cortical engagement in the P3 task, in the attempt to preserve both gait and cognitive performances. In HD patients the association with the cognitive tests produced only a slight and not relevant deterioration of motor speed and muscle recruitment, but the dual-task condition caused some modulation of EEG beta band activity. These findings suggest that combined cognitive and motor stimulation, in the form of dual-task conditions, could be used for rehabilitative purposes, as a means of enhancing the activation of compensatory cortical reserves and thus counteract potential interference between cognitive and motor processes and promote the integration of neuronal circuits serving different functions.

## Highlights

HD patients and controls performed an auditory P3 task whilst walking.

During walking P3 amplitude was higher and EEG rhythms were modulated in HD and controls.

Cortical activation may promote the integration of neuronal circuits serving different functions.

## Author contributions

Md: study design and coordination, data analysis, and manuscript preparation. KR and AM: EEG and EMG data recording, data analysis. EV: patients selection, data analysis. SI: data analysis, manuscript editing.

### Conflict of interest statement

The authors declare that the research was conducted in the absence of any commercial or financial relationships that could be construed as a potential conflict of interest.
