# Supplementary material for: Walking-Related Dual-Task Interference in Early-to-Middle-Stage Huntington's Disease: An Auditory Event Related Potential Study
Source: Front Psychol. 2017 Jul 31;8:1292. doi: 10.3389/fpsyg.2017.01292 (PMC5535504; doi:10.3389/fpsyg.2017.01292)
Supplement: Supplementary file 1 [file Table1.DOCX]

|  |  |  | MEAN | ERROR DS | 95% CI |  |
| --- | --- | --- | --- | --- | --- | --- |
|  |  |  |  |  | LOWER | UPPER |
| Fp1 | .CONTROLS | WALKING | 11230.19 | 2189.11 | 6848.21 | 15612.16 |
|  |  | STANDING | 2979.34 | 2098.17 | -1220.59 | 7179.28 |
|  | HD | WALKING | 7685.18 | 1672.41 | 4337.50 | 11032.87 |
|  |  | STANDING | 6381.35 | 2212.39 | 1952.78 | 10809.93 |
| Fpz | .CONTROLS | WALKING | 6578.55 | 1854.43 | 2866.51 | 10290.59 |
|  |  | STANDING | 2707.95 | 1777.39 | -849.88 | 6265.78 |
|  | HD | WALKING | 5951.46 | 1416.72 | 3115.59 | 8787.34 |
|  |  | STANDING | 4090.53 | 1874.15 | 339.01 | 7842.04 |
| Fp2 | .CONTROLS | WALKING | 7140.62 | 2649.11 | 1837.85 | 12443.39 |
|  |  | STANDING | 2836.53 | 2539.06 | -2245.95 | 7919.00 |
|  | HD | WALKING | 10381.71 | 2023.83 | 6330.57 | 14432.85 |
|  |  | STANDING | 6937.12 | 2677.28 | 1577.96 | 12296.28 |
| F7 | .CONTROLS | WALKING | 11385.82 | 1996.97 | 7388.45 | 15383.20 |
|  |  | STANDING | 4774.38 | 1914.01 | 943.07 | 8605.69 |
|  | HD | WALKING | 6975.55 | 1525.62 | 3921.68 | 10029.41 |
|  |  | STANDING | 3158.95 | 2018.21 | -880.94 | 7198.83 |
| F3 | .CONTROLS | WALKING | 9795.59 | 2207.84 | 5376.11 | 14215.06 |
|  |  | STANDING | 4372.16 | 2116.12 | 136.29 | 8608.04 |
|  | HD | WALKING | 4238.95 | 1686.72 | 862.62 | 7615.29 |
|  |  | STANDING | 675.28 | 2231.32 | -3791.19 | 5141.76 |
| Fz | .CONTROLS | WALKING | 8787.69 | 1523.69 | 5737.70 | 11837.68 |
|  |  | STANDING | 3531.10 | 1460.39 | 607.82 | 6454.38 |
|  | HD | WALKING | 6003.84 | 1164.05 | 3673.75 | 8333.93 |
|  |  | STANDING | 1758.93 | 1539.89 | -1323.50 | 4841.35 |
| F4 | .CONTROLS | WALKING | 6972.41 | 2131.23 | 2706.29 | 11238.54 |
|  |  | STANDING | 3793.14 | 2042.69 | -295.75 | 7882.04 |
|  | HD | WALKING | 8546.07 | 1628.19 | 5286.89 | 11805.25 |
|  |  | STANDING | 2451.62 | 2153.90 | -1859.87 | 6763.12 |
| F8 | .CONTROLS | WALKING | 11752.59 | 3550.04 | 4646.42 | 18858.76 |
|  |  | STANDING | 3229.54 | 3402.56 | -3581.42 | 10040.50 |
|  | HD | WALKING | 14239.03 | 2712.11 | 8810.14 | 19667.91 |
|  |  | STANDING | 7165.62 | 3587.79 | -16.12 | 14347.36 |
| T3 | .CONTROLS | WALKING | 8657.61 | 2143.39 | 4367.15 | 12948.07 |
|  |  | STANDING | 3455.19 | 2054.34 | -657.03 | 7567.40 |
|  | HD | WALKING | 5906.77 | 1637.48 | 2629.00 | 9184.54 |
|  |  | STANDING | 4535.04 | 2166.18 | 198.96 | 8871.13 |
| C3 | .CONTROLS | WALKING | 9690.83 | 1699.72 | 6288.48 | 13093.18 |
|  |  | STANDING | 3957.03 | 1629.10 | 696.02 | 7218.03 |
|  | HD | WALKING | 5419.83 | 1298.53 | 2820.54 | 8019.11 |
|  |  | STANDING | 4120.04 | 1717.79 | 681.51 | 7558.57 |
| Cz | .CONTROLS | WALKING | 8057.38 | 1835.57 | 4383.08 | 11731.68 |
|  |  | STANDING | 2975.31 | 1759.32 | -546.35 | 6496.96 |
|  | HD | WALKING | 9070.88 | 1402.32 | 6263.84 | 11877.93 |
|  |  | STANDING | 4413.75 | 1855.09 | 700.38 | 8127.12 |
| C4 | .CONTROLS | WALKING | 11614.59 | 3652.35 | 4303.62 | 18925.55 |
|  |  | STANDING | 2708.96 | 3500.62 | -4298.29 | 9716.20 |
|  | HD | WALKING | 12129.50 | 2790.28 | 6544.16 | 17714.84 |
|  |  | STANDING | 8713.48 | 3691.19 | 1324.77 | 16102.20 |
| T4 | .CONTROLS | WALKING | 9874.30 | 2528.82 | 4812.31 | 14936.29 |
|  |  | STANDING | 3219.18 | 2423.77 | -1632.51 | 8070.88 |
|  | HD | WALKING | 12297.97 | 1931.94 | 8430.78 | 16165.16 |
|  |  | STANDING | 8497.05 | 2555.71 | 3381.24 | 13612.87 |
| T5 | .CONTROLS | WALKING | 7672.57 | 2078.73 | 3511.54 | 11833.60 |
|  |  | STANDING | 2773.63 | 1992.37 | -1214.54 | 6761.80 |
|  | HD | WALKING | 6148.72 | 1588.08 | 2969.82 | 9327.61 |
|  |  | STANDING | 3350.08 | 2100.84 | -855.20 | 7555.36 |
| P3 | .CONTROLS | WALKING | 15291.43 | 3648.88 | 7987.40 | 22595.45 |
|  |  | STANDING | 3784.46 | 3497.29 | -3216.14 | 10785.05 |
|  | HD | WALKING | 9703.20 | 2787.63 | 4123.16 | 15283.24 |
|  |  | STANDING | 6706.52 | 3687.68 | -675.18 | 14088.22 |
| Pz | .CONTROLS | WALKING | 9969.70 | 2779.65 | 4405.63 | 15533.76 |
|  |  | STANDING | 3963.88 | 2664.17 | -1369.04 | 9296.79 |
|  | HD | WALKING | 10656.41 | 2123.56 | 6405.64 | 14907.17 |
|  |  | STANDING | 5186.00 | 2809.20 | -437.24 | 10809.23 |
| P4 | .CONTROLS | WALKING | 9090.18 | 2305.31 | 4475.61 | 13704.76 |
|  |  | STANDING | 4161.67 | 2209.54 | -261.20 | 8584.54 |
|  | HD | WALKING | 8828.18 | 1761.18 | 5302.79 | 12353.56 |
|  |  | STANDING | 5243.24 | 2329.82 | 579.60 | 9906.89 |
| T6 | .CONTROLS | WALKING | 10395.22 | 2697.07 | 4996.44 | 15793.99 |
|  |  | STANDING | 2605.35 | 2585.03 | -2569.14 | 7779.84 |
|  | HD | WALKING | 7524.55 | 2060.48 | 3400.06 | 11649.04 |
|  |  | STANDING | 4222.51 | 2725.75 | -1233.68 | 9678.70 |
| O1 | .CONTROLS | WALKING | 10321.12 | 2748.58 | 4819.23 | 15823.01 |
|  |  | STANDING | 3278.59 | 2634.40 | -1994.73 | 8551.91 |
|  | HD | WALKING | 6004.89 | 2099.83 | 1801.62 | 10208.15 |
|  |  | STANDING | 4636.54 | 2777.81 | -923.86 | 10196.94 |
| Oz | .CONTROLS | WALKING | 14134.02 | 2331.07 | 9467.87 | 18800.17 |
|  |  | STANDING | 3072.37 | 2234.23 | -1399.93 | 7544.67 |
|  | HD | WALKING | 10739.44 | 1780.86 | 7174.66 | 14304.23 |
|  |  | STANDING | 3753.04 | 2355.86 | -962.73 | 8468.81 |
| O2 | .CONTROLS | WALKING | 10033.55 | 2719.05 | 4590.78 | 15476.31 |
|  |  | STANDING | 3258.84 | 2606.09 | -1957.81 | 8475.49 |
|  | HD | WALKING | 7557.41 | 2077.26 | 3399.32 | 11715.51 |
|  |  | STANDING | 6572.26 | 2747.96 | 1071.61 | 12072.90 |
|  | | | | | | |

Table S1 Mean values and standard errors of P3 amplitudes by the target acoustic stimulus in HD patients and controls. The values are expressed in nanoVolts, as originally provided by ASA software. a. Regression with weighted least squares method - Weighted by AGE

|  | CONTROLS | |  | HD PATIENTS | |  |
| --- | --- | --- | --- | --- | --- | --- |
|  | T | df | Sig. (2-code) | t | df | Sig. (2-code) |
|  |  | 26 |  |  | 48 |  |
| Fp1 | -3.15 |  | 0.00 | -0.63 |  | 0.53 |
| Fpz | -2.42 |  | 0.02 | -0.77 |  | 0.45 |
| Fp2 | -4.63 |  | 0.00 | -1.33 |  | 0.19 |
| F7 | -2.93 |  | 0.01 | -1.91 |  | 0.06 |
| F3 | -1.84 |  | 0.08 | -1.20 |  | 0.24 |
| Fz | -2.67 |  | 0.01 | -2.99 |  | 0.00 |
| F4 | -2.28 |  | 0.03 | -1.56 |  | 0.13 |
| F8 | -1.77 |  | 0.09 | -2.36 |  | 0.02 |
| T3 | -2.39 |  | 0.02 | -0.81 |  | 0.42 |
| C3 | -2.36 |  | 0.03 | -0.95 |  | 0.35 |
| Cz | -2.51 |  | 0.02 | -2.56 |  | 0.01 |
| C4 | -2.20 |  | 0.04 | -1.23 |  | 0.22 |
| T4 | -1.95 |  | 0.06 | -2.72 |  | 0.01 |
| T5 | -2.25 |  | 0.03 | -1.46 |  | 0.15 |
| P3 | -2.20 |  | 0.04 | -1.62 |  | 0.11 |
| Pz | -2.32 |  | 0.03 | -2.76 |  | 0.01 |
| P4 | -1.86 |  | 0.07 | -2.36 |  | 0.02 |
| T6 | -2.09 |  | 0.05 | -1.83 |  | 0.07 |
| O1 | -1.72 |  | 0.10 | -1.89 |  | 0.07 |
| Oz | -3.18 |  | 0.00 | -4.36 |  | 0.00 |
| O2 | -2.16 |  | 0.04 | -1.85 |  | 0.07 |

Table S2 Students’ t test for paired data , corrected for multiple comparisons between P3 in standing and walking condition in HD patients and controls.

| VARIABLES | | | Mean | DS | 95% | |
| --- | --- | --- | --- | --- | --- | --- |
|  |  |  |  |  | LOWER | UPPER |
| fp1log | CONTROLS | WALKING | 5.325 | .165 | 4.999 | 5.652 |
|  |  | STANDING | 4.286 | .165 | 3.960 | 4.612 |
|  |  | P3 WALKING | 5.305 | .189 | 4.932 | 5.677 |
|  |  | P3 STANDING | 4.218 | .165 | 3.892 | 4.544 |
|  | HD | WALKING | 5.127 | .124 | 4.882 | 5.372 |
|  |  | STANDING | 4.737 | .124 | 4.492 | 4.983 |
|  |  | P3 WALKING | 5.201 | .126 | 4.951 | 5.451 |
|  |  | P3 STANDING | 4.767 | .126 | 4.517 | 5.017 |
| fpzlog | CONTROLS | WALKING | 5.172 | .163 | 4.850 | 5.494 |
|  |  | STANDING | 4.122 | .163 | 3.800 | 4.444 |
|  |  | P3 WALKING | 5.037 | .186 | 4.670 | 5.405 |
|  |  | P3 STANDING | 4.164 | .163 | 3.842 | 4.486 |
|  | HD | WALKING | 5.183 | .123 | 4.941 | 5.426 |
|  |  | STANDING | 4.450 | .123 | 4.208 | 4.693 |
|  |  | P3 WALKING | 5.171 | .125 | 4.924 | 5.418 |
|  |  | P3 STANDING | 4.559 | .125 | 4.312 | 4.805 |
| fp2log | CONTROLS | WALKING | 5.348 | .139 | 5.073 | 5.623 |
|  |  | STANDING | 4.293 | .139 | 4.018 | 4.568 |
|  |  | P3 WALKING | 5.340 | .159 | 5.026 | 5.654 |
|  |  | P3 STANDING | 4.214 | .139 | 3.939 | 4.488 |
|  | HD | WALKING | 5.270 | .105 | 5.064 | 5.477 |
|  |  | STANDING | 4.476 | .105 | 4.269 | 4.683 |
|  |  | P3 WALKING | 5.110 | .107 | 4.899 | 5.320 |
|  |  | P3 STANDING | 4.691 | .107 | 4.481 | 4.902 |
| f7log | CONTROLS | WALKING | 5.699 | .116 | 5.471 | 5.928 |
|  |  | STANDING | 4.809 | .116 | 4.581 | 5.038 |
|  |  | P3 WALKING | 5.648 | .132 | 5.387 | 5.909 |
|  |  | P3 STANDING | 4.597 | .116 | 4.368 | 4.825 |
|  | HD | WALKING | 5.153 | .087 | 4.981 | 5.325 |
|  |  | STANDING | 4.779 | .087 | 4.607 | 4.951 |
|  |  | P3 WALKING | 5.234 | .089 | 5.058 | 5.409 |
|  |  | P3 STANDING | 4.849 | .089 | 4.674 | 5.024 |
| f3log | CONTROLS | WALKING | 5.785 | .140 | 5.508 | 6.062 |
|  |  | STANDING | 4.507 | .140 | 4.230 | 4.785 |
|  |  | P3 WALKING | 5.524 | .160 | 5.207 | 5.841 |
|  |  | P3 STANDING | 4.432 | .140 | 4.155 | 4.709 |
|  | HD | WALKING | 5.084 | .106 | 4.875 | 5.292 |
|  |  | STANDING | 4.562 | .106 | 4.353 | 4.771 |
|  |  | P3 WALKING | 5.213 | .108 | 5.001 | 5.426 |
|  |  | P3 STANDING | 4.592 | .108 | 4.380 | 4.805 |
| fzlog | CONTROLS | WALKING | 5.643 | .135 | 5.376 | 5.910 |
|  |  | STANDING | 4.379 | .135 | 4.112 | 4.646 |
|  |  | P3 WALKING | 5.506 | .154 | 5.201 | 5.811 |
|  |  | P3 STANDING | 4.426 | .135 | 4.159 | 4.693 |
|  | HD | WALKING | 4.999 | .102 | 4.798 | 5.200 |
|  |  | STANDING | 4.489 | .102 | 4.288 | 4.689 |
|  |  | P3 WALKING | 5.028 | .103 | 4.823 | 5.232 |
|  |  | P3 STANDING | 4.583 | .103 | 4.378 | 4.788 |
| f4log | CONTROLS | WALKING | 5.718 | .136 | 5.449 | 5.987 |
|  |  | STANDING | 4.518 | .136 | 4.249 | 4.787 |
|  |  | P3 WALKING | 5.379 | .156 | 5.071 | 5.686 |
|  |  | P3 STANDING | 4.538 | .136 | 4.269 | 4.808 |
|  | HD | WALKING | 5.077 | .102 | 4.874 | 5.279 |
|  |  | STANDING | 4.584 | .102 | 4.381 | 4.786 |
|  |  | P3 WALKING | 5.044 | .104 | 4.838 | 5.251 |
|  |  | P3 STANDING | 4.642 | .104 | 4.436 | 4.848 |
| f8log | CONTROLS | WALKING | 5.850 | .119 | 5.615 | 6.084 |
|  |  | STANDING | 4.689 | .119 | 4.454 | 4.924 |
|  |  | P3 WALKING | 5.650 | .136 | 5.382 | 5.918 |
|  |  | P3 STANDING | 4.540 | .119 | 4.305 | 4.775 |
|  | HD | WALKING | 5.343 | .089 | 5.167 | 5.520 |
|  |  | STANDING | 4.770 | .089 | 4.593 | 4.946 |
|  |  | P3 WALKING | 5.292 | .091 | 5.112 | 5.472 |
|  |  | P3 STANDING | 4.889 | .091 | 4.709 | 5.069 |
| t3log | CONTROLS | WALKING | 5.716 | .139 | 5.441 | 5.992 |
|  |  | STANDING | 4.576 | .139 | 4.301 | 4.851 |
|  |  | P3 WALKING | 5.410 | .159 | 5.095 | 5.724 |
|  |  | P3 STANDING | 4.381 | .139 | 4.106 | 4.656 |
|  | HD | WALKING | 5.243 | .105 | 5.035 | 5.450 |
|  |  | STANDING | 4.735 | .105 | 4.528 | 4.942 |
|  |  | P3 WALKING | 5.195 | .107 | 4.984 | 5.406 |
|  |  | P3 STANDING | 4.705 | .107 | 4.494 | 4.916 |
| c3log | CONTROLS | WALKING | 5.800 | .150 | 5.502 | 6.097 |
|  |  | STANDING | 4.545 | .150 | 4.248 | 4.842 |
|  |  | P3 WALKING | 5.636 | .172 | 5.297 | 5.976 |
|  |  | P3 STANDING | 4.601 | .150 | 4.304 | 4.898 |
|  | HD | WALKING | 5.046 | .113 | 4.822 | 5.269 |
|  |  | STANDING | 4.615 | .113 | 4.392 | 4.839 |
|  |  | P3 WALKING | 5.027 | .115 | 4.799 | 5.254 |
|  |  | P3 STANDING | 4.655 | .115 | 4.428 | 4.883 |
| czlog | CONTROLS | WALKING | 5.735 | .151 | 5.436 | 6.033 |
|  |  | STANDING | 4.568 | .151 | 4.269 | 4.866 |
|  |  | P3 WALKING | 5.491 | .173 | 5.150 | 5.833 |
|  |  | P3 STANDING | 4.512 | .151 | 4.213 | 4.811 |
|  | HD | WALKING | 5.065 | .114 | 4.840 | 5.289 |
|  |  | STANDING | 4.488 | .114 | 4.263 | 4.713 |
|  |  | P3 WALKING | 5.082 | .116 | 4.854 | 5.311 |
|  |  | P3 STANDING | 4.566 | .116 | 4.337 | 4.795 |
| c4log | CONTROLS | WALKING | 5.780 | .163 | 5.457 | 6.102 |
|  |  | STANDING | 4.437 | .163 | 4.114 | 4.759 |
|  |  | P3 WALKING | 5.505 | .186 | 5.136 | 5.873 |
|  |  | P3 STANDING | 4.414 | .163 | 4.091 | 4.736 |
|  | HD | WALKING | 5.176 | .123 | 4.934 | 5.419 |
|  |  | STANDING | 4.545 | .123 | 4.302 | 4.788 |
|  |  | P3 WALKING | 5.282 | .125 | 5.035 | 5.529 |
|  |  | P3 STANDING | 4.579 | .125 | 4.332 | 4.826 |
| t4log | CONTROLS | WALKING | 5.774 | .126 | 5.525 | 6.023 |
|  |  | STANDING | 4.621 | .126 | 4.372 | 4.869 |
|  |  | P3 WALKING | 5.542 | .144 | 5.257 | 5.826 |
|  |  | P3 STANDING | 4.501 | .126 | 4.252 | 4.750 |
|  | HD | WALKING | 5.179 | .095 | 4.992 | 5.366 |
|  |  | STANDING | 4.656 | .095 | 4.469 | 4.843 |
|  |  | P3 WALKING | 5.118 | .096 | 4.927 | 5.309 |
|  |  | P3 STANDING | 4.652 | .096 | 4.461 | 4.842 |
| t5log | CONTROLS | WALKING | 5.821 | .142 | 5.541 | 6.102 |
|  |  | STANDING | 4.597 | .142 | 4.317 | 4.878 |
|  |  | P3 WALKING | 5.683 | .162 | 5.362 | 6.003 |
|  |  | P3 STANDING | 4.562 | .142 | 4.281 | 4.842 |
|  | HD | WALKING | 5.089 | .107 | 4.878 | 5.300 |
|  |  | STANDING | 4.614 | .107 | 4.403 | 4.825 |
|  |  | P3 WALKING | 5.126 | .109 | 4.911 | 5.341 |
|  |  | P3 STANDING | 4.602 | .109 | 4.387 | 4.816 |
| p3log | CONTROLS | WALKING | 5.839 | .160 | 5.522 | 6.155 |
|  |  | STANDING | 4.422 | .160 | 4.105 | 4.739 |
|  |  | P3 WALKING | 5.787 | .183 | 5.426 | 6.149 |
|  |  | P3 STANDING | 4.496 | .160 | 4.179 | 4.813 |
|  | HD | WALKING | 5.315 | .121 | 5.076 | 5.553 |
|  |  | STANDING | 4.559 | .121 | 4.321 | 4.798 |
|  |  | P3 WALKING | 5.115 | .123 | 4.872 | 5.358 |
|  |  | P3 STANDING | 4.688 | .123 | 4.445 | 4.931 |
| pzlog | CONTROLS | WALKING | 5.784 | .179 | 5.429 | 6.138 |
|  |  | STANDING | 4.486 | .179 | 4.131 | 4.840 |
|  |  | P3 WALKING | 5.565 | .205 | 5.159 | 5.970 |
|  |  | P3 STANDING | 4.498 | .179 | 4.143 | 4.852 |
|  | HD | WALKING | 5.131 | .135 | 4.864 | 5.398 |
|  |  | STANDING | 4.412 | .135 | 4.145 | 4.679 |
|  |  | P3 WALKING | 5.166 | .138 | 4.894 | 5.438 |
|  |  | P3 STANDING | 4.556 | .138 | 4.284 | 4.828 |
| p4log | CONTROLS | WALKING | 5.731 | .166 | 5.404 | 6.058 |
|  |  | STANDING | 4.446 | .166 | 4.118 | 4.773 |
|  |  | P3 WALKING | 5.625 | .189 | 5.251 | 5.999 |
|  |  | P3 STANDING | 4.512 | .166 | 4.185 | 4.840 |
|  | HD | WALKING | 4.996 | .125 | 4.750 | 5.242 |
|  |  | STANDING | 4.453 | .125 | 4.207 | 4.700 |
|  |  | P3 WALKING | 5.027 | .127 | 4.777 | 5.278 |
|  |  | P3 STANDING | 4.537 | .127 | 4.286 | 4.788 |
| t6log | CONTROLS | WALKING | 5.662 | .136 | 5.393 | 5.930 |
|  |  | STANDING | 4.609 | .136 | 4.341 | 4.878 |
|  |  | P3 WALKING | 5.567 | .155 | 5.260 | 5.873 |
|  |  | P3 STANDING | 4.630 | .136 | 4.362 | 4.899 |
|  | HD | WALKING | 5.120 | .102 | 4.918 | 5.322 |
|  |  | STANDING | 4.606 | .102 | 4.404 | 4.808 |
|  |  | P3 WALKING | 5.075 | .104 | 4.869 | 5.280 |
|  |  | P3 STANDING | 4.653 | .104 | 4.448 | 4.859 |
| o1log | CONTROLS | WALKING | 5.822 | .133 | 5.558 | 6.086 |
|  |  | STANDING | 4.647 | .133 | 4.383 | 4.910 |
|  |  | P3 WALKING | 5.796 | .152 | 5.495 | 6.097 |
|  |  | P3 STANDING | 4.645 | .133 | 4.381 | 4.909 |
|  | HD | WALKING | 5.280 | .100 | 5.082 | 5.479 |
|  |  | STANDING | 4.674 | .100 | 4.476 | 4.873 |
|  |  | P3 WALKING | 5.246 | .102 | 5.044 | 5.448 |
|  |  | P3 STANDING | 4.764 | .102 | 4.562 | 4.966 |
| ozlog | CONTROLS | WALKING | 5.977 | .147 | 5.686 | 6.268 |
|  |  | STANDING | 4.685 | .147 | 4.394 | 4.976 |
|  |  | P3 WALKING | 5.837 | .168 | 5.505 | 6.169 |
|  |  | P3 STANDING | 4.570 | .147 | 4.280 | 4.861 |
|  | HD | WALKING | 5.194 | .111 | 4.976 | 5.413 |
|  |  | STANDING | 4.698 | .111 | 4.479 | 4.917 |
|  |  | P3 WALKING | 5.166 | .113 | 4.943 | 5.389 |
|  |  | P3 STANDING | 4.746 | .113 | 4.523 | 4.969 |
| o2log | CONTROLS | WALKING | 5.885 | .140 | 5.608 | 6.162 |
|  |  | STANDING | 4.661 | .140 | 4.384 | 4.937 |
|  |  | P3 WALKING | 5.885 | .160 | 5.569 | 6.202 |
|  |  | P3 STANDING | 4.642 | .140 | 4.365 | 4.919 |
|  | HD | WALKING | 5.294 | .105 | 5.086 | 5.502 |
|  |  | STANDING | 4.678 | .105 | 4.470 | 4.887 |
|  |  | P3 WALKING | 5.177 | .107 | 4.965 | 5.389 |
|  |  | P3 STANDING | 4.753 | .107 | 4.540 | 4.965 |
| Table S3 Mean values of alpha rhythm (log) in HD patients and controls  a. Regression with weighted least squares method - Weighted by AGE | | | | | | |

|  | | | Mean | DS | 95% CI | |
| --- | --- | --- | --- | --- | --- | --- |
|  |  |  |  |  | LOWER | HIGHER |
| lfp1 | CONTROLS | WALKING | 4.947 | .215 | 4.520 | 5.374 |
|  |  | STANDING | 4.585 | .142 | 4.304 | 4.867 |
|  |  | P3 WALKING | 4.820 | .287 | 4.251 | 5.390 |
|  |  | P3 STANDING | 4.657 | .142 | 4.376 | 4.939 |
|  | HD | WALKING | 4.876 | .154 | 4.571 | 5.182 |
|  |  | STANDING | 4.997 | .112 | 4.775 | 5.219 |
|  |  | P3 WALKING | 5.026 | .137 | 4.754 | 5.299 |
|  |  | P3 STANDING | 4.869 | .119 | 4.633 | 5.105 |
| lfpz | CONTROLS | WALKING | 4.567 | .159 | 4.251 | 4.882 |
|  |  | STANDING | 4.257 | .105 | 4.049 | 4.465 |
|  |  | P3 WALKING | 4.596 | .212 | 4.175 | 5.017 |
|  |  | P3 STANDING | 4.308 | .105 | 4.100 | 4.516 |
|  | HD | WALKING | 4.722 | .114 | 4.496 | 4.948 |
|  |  | STANDING | 4.488 | .083 | 4.324 | 4.652 |
|  |  | P3 WALKING | 4.860 | .101 | 4.658 | 5.061 |
|  |  | P3 STANDING | 4.545 | .088 | 4.371 | 4.720 |
| lfp2 | CONTROLS | WALKING | 4.889 | .183 | 4.525 | 5.254 |
|  |  | STANDING | 4.620 | .121 | 4.380 | 4.860 |
|  |  | P3 WALKING | 5.064 | .244 | 4.579 | 5.550 |
|  |  | P3 STANDING | 4.691 | .121 | 4.450 | 4.931 |
|  | HD | WALKING | 4.916 | .131 | 4.656 | 5.177 |
|  |  | STANDING | 4.701 | .095 | 4.511 | 4.890 |
|  |  | P3 WALKING | 4.889 | .117 | 4.657 | 5.121 |
|  |  | P3 STANDING | 4.739 | .101 | 4.538 | 4.940 |
| lf7 | CONTROLS | WALKING | 5.204 | .144 | 4.917 | 5.490 |
|  |  | STANDING | 4.954 | .095 | 4.765 | 5.143 |
|  |  | P3 WALKING | 5.211 | .192 | 4.829 | 5.593 |
|  |  | P3 STANDING | 4.760 | .095 | 4.571 | 4.949 |
|  | HD | WALKING | 4.920 | .103 | 4.715 | 5.125 |
|  |  | STANDING | 4.932 | .075 | 4.783 | 5.081 |
|  |  | P3 WALKING | 5.079 | .092 | 4.896 | 5.261 |
|  |  | P3 STANDING | 4.892 | .080 | 4.734 | 5.050 |
| lf3 | CONTROLS | WALKING | 5.060 | .144 | 4.774 | 5.346 |
|  |  | STANDING | 4.796 | .095 | 4.607 | 4.984 |
|  |  | P3 WALKING | 4.780 | .192 | 4.398 | 5.161 |
|  |  | P3 STANDING | 4.691 | .095 | 4.503 | 4.880 |
|  | HD | WALKING | 4.919 | .103 | 4.714 | 5.123 |
|  |  | STANDING | 4.735 | .075 | 4.587 | 4.884 |
|  |  | P3 WALKING | 5.033 | .092 | 4.851 | 5.215 |
|  |  | P3 STANDING | 4.761 | .080 | 4.603 | 4.919 |
| lfz | CONTROLS | WALKING | 4.841 | .123 | 4.597 | 5.084 |
|  |  | STANDING | 4.506 | .081 | 4.346 | 4.667 |
|  |  | P3 WALKING | 4.899 | .163 | 4.575 | 5.224 |
|  |  | P3 STANDING | 4.422 | .081 | 4.261 | 4.582 |
|  | HD | WALKING | 4.656 | .088 | 4.481 | 4.830 |
|  |  | STANDING | 4.517 | .064 | 4.391 | 4.644 |
|  |  | P3 WALKING | 4.766 | .078 | 4.610 | 4.921 |
|  |  | P3 STANDING | 4.505 | .068 | 4.370 | 4.639 |
| lf4 | CONTROLS | WALKING | 4.989 | .130 | 4.730 | 5.248 |
|  |  | STANDING | 4.709 | .086 | 4.538 | 4.879 |
|  |  | P3 WALKING | 4.858 | .174 | 4.513 | 5.203 |
|  |  | P3 STANDING | 4.659 | .086 | 4.488 | 4.830 |
|  | HD | WALKING | 4.842 | .093 | 4.657 | 5.027 |
|  |  | STANDING | 4.751 | .068 | 4.616 | 4.885 |
|  |  | P3 WALKING | 4.894 | .083 | 4.729 | 5.059 |
|  |  | P3 STANDING | 4.726 | .072 | 4.583 | 4.868 |
| lf8 | CONTROLS | WALKING | 5.009 | .129 | 4.753 | 5.266 |
|  |  | STANDING | 4.803 | .085 | 4.633 | 4.972 |
|  |  | P3 WALKING | 4.868 | .172 | 4.526 | 5.210 |
|  |  | P3 STANDING | 4.691 | .085 | 4.522 | 4.860 |
|  | HD | WALKING | 5.050 | .092 | 4.866 | 5.233 |
|  |  | STANDING | 4.928 | .067 | 4.794 | 5.061 |
|  |  | P3 WALKING | 5.110 | .082 | 4.946 | 5.273 |
|  |  | P3 STANDING | 4.890 | .071 | 4.748 | 5.031 |
| lt3 | CONTROLS | WALKING | 4.983 | .130 | 4.724 | 5.243 |
|  |  | STANDING | 4.819 | .086 | 4.648 | 4.990 |
|  |  | P3 WALKING | 4.871 | .174 | 4.526 | 5.217 |
|  |  | P3 STANDING | 4.706 | .086 | 4.535 | 4.877 |
|  | HD | WALKING | 4.964 | .093 | 4.779 | 5.150 |
|  |  | STANDING | 4.885 | .068 | 4.751 | 5.020 |
|  |  | P3 WALKING | 5.055 | .083 | 4.890 | 5.220 |
|  |  | P3 STANDING | 4.891 | .072 | 4.747 | 5.034 |
| lc3 | CONTROLS | WALKING | 4.993 | .140 | 4.715 | 5.271 |
|  |  | STANDING | 4.713 | .092 | 4.530 | 4.896 |
|  |  | P3 WALKING | 4.754 | .186 | 4.384 | 5.125 |
|  |  | P3 STANDING | 4.655 | .092 | 4.472 | 4.838 |
|  | HD | WALKING | 4.570 | .100 | 4.371 | 4.769 |
|  |  | STANDING | 4.597 | .073 | 4.452 | 4.741 |
|  |  | P3 WALKING | 4.749 | .089 | 4.572 | 4.926 |
|  |  | P3 STANDING | 4.691 | .077 | 4.538 | 4.845 |
| lcz | CONTROLS | WALKING | 4.533 | .119 | 4.297 | 4.768 |
|  |  | STANDING | 4.593 | .078 | 4.437 | 4.748 |
|  |  | P3 WALKING | 4.589 | .158 | 4.275 | 4.903 |
|  |  | P3 STANDING | 4.465 | .078 | 4.309 | 4.620 |
|  | HD | WALKING | 4.442 | .085 | 4.274 | 4.611 |
|  |  | STANDING | 4.464 | .062 | 4.342 | 4.587 |
|  |  | P3 WALKING | 4.618 | .076 | 4.468 | 4.768 |
|  |  | P3 STANDING | 4.432 | .065 | 4.302 | 4.562 |
| lc4 | CONTROLS | WALKING | 4.690 | .144 | 4.403 | 4.977 |
|  |  | STANDING | 4.655 | .095 | 4.466 | 4.845 |
|  |  | P3 WALKING | 4.718 | .193 | 4.336 | 5.101 |
|  |  | P3 STANDING | 4.500 | .095 | 4.310 | 4.689 |
|  | HD | WALKING | 4.744 | .103 | 4.539 | 4.950 |
|  |  | STANDING | 4.665 | .075 | 4.516 | 4.814 |
|  |  | P3 WALKING | 4.982 | .092 | 4.799 | 5.165 |
|  |  | P3 STANDING | 4.637 | .080 | 4.478 | 4.795 |
| lt4 | CONTROLS | WALKING | 4.981 | .126 | 4.730 | 5.231 |
|  |  | STANDING | 4.830 | .083 | 4.665 | 4.994 |
|  |  | P3 WALKING | 4.750 | .168 | 4.417 | 5.084 |
|  |  | P3 STANDING | 4.747 | .083 | 4.582 | 4.912 |
|  | HD | WALKING | 4.993 | .090 | 4.814 | 5.172 |
|  |  | STANDING | 4.875 | .065 | 4.745 | 5.005 |
|  |  | P3 WALKING | 5.034 | .080 | 4.874 | 5.193 |
|  |  | P3 STANDING | 4.811 | .070 | 4.673 | 4.949 |
| lt5 | CONTROLS | WALKING | 4.966 | .130 | 4.708 | 5.225 |
|  |  | STANDING | 4.884 | .086 | 4.714 | 5.054 |
|  |  | P3 WALKING | 4.913 | .174 | 4.569 | 5.258 |
|  |  | P3 STANDING | 4.692 | .086 | 4.521 | 4.863 |
|  | HD | WALKING | 4.817 | .093 | 4.632 | 5.002 |
|  |  | STANDING | 4.731 | .068 | 4.596 | 4.865 |
|  |  | P3 WALKING | 4.999 | .083 | 4.835 | 5.164 |
|  |  | P3 STANDING | 4.651 | .072 | 4.508 | 4.794 |
| lp3 | CONTROLS | WALKING | 4.836 | .174 | 4.491 | 5.181 |
|  |  | STANDING | 4.595 | .114 | 4.368 | 4.823 |
|  |  | P3 WALKING | 4.851 | .231 | 4.391 | 5.311 |
|  |  | P3 STANDING | 4.589 | .115 | 4.362 | 4.817 |
|  | HD | WALKING | 4.687 | .124 | 4.440 | 4.934 |
|  |  | STANDING | 4.542 | .090 | 4.362 | 4.721 |
|  |  | P3 WALKING | 4.804 | .111 | 4.584 | 5.024 |
|  |  | P3 STANDING | 4.570 | .096 | 4.380 | 4.761 |
| lpz | CONTROLS | WALKING | 4.458 | .140 | 4.180 | 4.736 |
|  |  | STANDING | 4.588 | .092 | 4.405 | 4.772 |
|  |  | P3 WALKING | 4.608 | .187 | 4.237 | 4.978 |
|  |  | P3 STANDING | 4.548 | .092 | 4.364 | 4.731 |
|  | HD | WALKING | 4.365 | .100 | 4.166 | 4.564 |
|  |  | STANDING | 4.363 | .073 | 4.219 | 4.508 |
|  |  | P3 WALKING | 4.644 | .089 | 4.467 | 4.822 |
|  |  | P3 STANDING | 4.413 | .077 | 4.259 | 4.566 |
| lp4 | CONTROLS | WALKING | 4.750 | .127 | 4.497 | 5.002 |
|  |  | STANDING | 4.695 | .084 | 4.528 | 4.861 |
|  |  | P3 WALKING | 4.882 | .169 | 4.545 | 5.219 |
|  |  | P3 STANDING | 4.633 | .084 | 4.467 | 4.800 |
|  | HD | WALKING | 4.496 | .091 | 4.315 | 4.677 |
|  |  | STANDING | 4.481 | .066 | 4.349 | 4.612 |
|  |  | P3 WALKING | 4.738 | .081 | 4.577 | 4.899 |
|  |  | P3 STANDING | 4.469 | .070 | 4.329 | 4.608 |
| lt6 | CONTROLS | WALKING | 4.930 | .111 | 4.710 | 5.150 |
|  |  | STANDING | 4.882 | .073 | 4.737 | 5.027 |
|  |  | P3 WALKING | 4.937 | .148 | 4.644 | 5.231 |
|  |  | P3 STANDING | 4.772 | .073 | 4.627 | 4.917 |
|  | HD | WALKING | 4.856 | .079 | 4.698 | 5.013 |
|  |  | STANDING | 4.715 | .058 | 4.601 | 4.830 |
|  |  | P3 WALKING | 4.999 | .071 | 4.859 | 5.140 |
|  |  | P3 STANDING | 4.673 | .061 | 4.551 | 4.794 |
| lo1 | CONTROLS | WALKING | 5.363 | .125 | 5.115 | 5.610 |
|  |  | STANDING | 4.789 | .082 | 4.625 | 4.952 |
|  |  | P3 WALKING | 4.994 | .166 | 4.664 | 5.324 |
|  |  | P3 STANDING | 4.703 | .082 | 4.540 | 4.866 |
|  | HD | WALKING | 4.900 | .089 | 4.723 | 5.077 |
|  |  | STANDING | 4.712 | .065 | 4.584 | 4.841 |
|  |  | P3 WALKING | 5.048 | .079 | 4.891 | 5.206 |
|  |  | P3 STANDING | 4.722 | .069 | 4.585 | 4.859 |
|  |  |  |  |  |  |  |
| loz | CONTROLS | WALKING | 5.055 | .132 | 4.793 | 5.318 |
|  |  | STANDING | 4.723 | .087 | 4.550 | 4.896 |
|  |  | P3 WALKING | 4.951 | .176 | 4.600 | 5.301 |
|  |  | P3 STANDING | 4.663 | .087 | 4.490 | 4.836 |
|  | HD | WALKING | 4.815 | .095 | 4.627 | 5.003 |
|  |  | STANDING | 4.640 | .069 | 4.504 | 4.777 |
|  |  | P3 WALKING | 4.965 | .084 | 4.798 | 5.132 |
|  |  | P3 STANDING | 4.653 | .073 | 4.507 | 4.798 |
| lo2 | CONTROLS | WALKING | 5.251 | .133 | 4.986 | 5.515 |
|  |  | STANDING | 4.843 | .088 | 4.668 | 5.017 |
|  |  | P3 WALKING | 5.206 | .178 | 4.853 | 5.559 |
|  |  | P3 STANDING | 4.831 | .088 | 4.657 | 5.006 |
|  | HD | WALKING | 4.787 | .095 | 4.597 | 4.976 |
|  |  | STANDING | 4.670 | .069 | 4.532 | 4.807 |
|  |  | P3 WALKING | 4.971 | .085 | 4.802 | 5.140 |
|  |  | P3 STANDING | 4.683 | .074 | 4.537 | 4.830 |

Table S4 Mean values of beta rhythms in Huntington’s Disease (HD) patients and controls a. Regression with weighted least squares method. Weighted for age.

| RIGTH ANTERIOR TIBIAL  Walking | | | df | | | F | | Sig. |
| --- | --- | --- | --- | --- | --- | --- | --- | --- |
|  |  | | 23 | | | **2.87** | | **.049** |
|  |  | |  | | |  | |  |
| Walking P3 | | |  | | |  | |  |
|  |  | | 23 | | | 1.451 | | .261^d^ |
| Walking | | t | | | Sig. | |  |  |
|  |  |  |  |  |  |  |  |  |
|  | CAG | -.612 | | | .551 | |  |  |
|  | DURATION | **3.366** | | | **.005** | |  |  |
|  | UHDRS | -.137 | | | .893 | |  |  |
|  | CHIOREA | -.839 | | | .417 | |  |  |
|  | CHOREA  LOWER LIMBS | .548 | | | .593 | |  |  |
|  | BRADIKINESA | .442 | | | .666 | |  |  |
|  | DISTONYA | .441 | | | .666 | |  |  |
|  | DISTONYA LOWER LIMBS | -.029 | | | .977 | |  |  |
|  | WALKING | -.242 | | | .812 | |  |  |
|  | TANDEM WALKING | .148 | | | .884 | |  |  |
| Walking P3 | | t | | | Sig. | |  |  |
|  |  |  |  |  |  |  |  |  |
|  | CAG | .099 | | | .923 | |  |  |
|  | DURATION | 1.524 | | | .151 | |  |  |
|  | UHDRSM | 1.587 | | | .136 | |  |  |
|  | CHOREA | -.421 | | | .681 | |  |  |
|  | CHOREA LOWER LIMBS | -1.904 | | | .079 | |  |  |
|  | BRADIKINESIA | -1.944 | | | .074 | |  |  |
|  | DISTONYA | -.210 | | | .837 | |  |  |
|  | DISTONYA LOWER LIMBS | -.540 | | | .598 | |  |  |
|  | WALKING | -1.241 | | | .236 | |  |  |
|  | TANDEM WALKING | 1.232 | | | .240 | |  |  |
| Walking  LEFT  ANTERIOR TIBIAL | | | df | | | F | | Sig. |
|  |  | |  | | | .581 | | .803^d^ |
|  | Total | | 23 | | |  | |  |
| Walking P3 | | | df | | | F | | Sig. |
|  |  | |  | | | .367 | | .941^d^ |
|  |  | | 23 | | |  | |  |
| Walking | |  |  |  |  |  |  |  |
|  |  | t | | | p | |  |  |
|  | CAG | -.206 | | | .840 | |  |  |
|  | DURATION | -.510 | | | .619 | |  |  |
|  | UHDRSm | .405 | | | .692 | |  |  |
|  | CHIOREA | -.145 | | | .887 | |  |  |
|  | CHOREA LOWER LIMBS | .565 | | | .581 | |  |  |
|  | BRADIKINESIA | .370 | | | .718 | |  |  |
|  | DISTONYS | -.099 | | | .922 | |  |  |
|  | DISTONYA LOWER LIMBS | .016 | | | .987 | |  |  |
|  | WALKING | -1.081 | | | .299 | |  |  |
|  | TANDEM WALKING | -.275 | | | .788 | |  |  |
| Walking P3 | | t | | | Sig. | |  |  |
|  |  |  |  |  |  |  |  |  |
| 1 | (Costante) | .682 | | | .507 | |  |  |
|  | CAG | -.140 | | | .891 | |  |  |
|  | DURATION | -.406 | | | .691 | |  |  |
|  | UHDRSm | .769 | | | .456 | |  |  |
|  | CHIOREA | -.551 | | | .591 | |  |  |
|  | CHOREA LOWER LIMBS | .497 | | | .627 | |  |  |
|  | BRADIKINESIA | .088 | | | .931 | |  |  |
|  | DISTONYS | -.337 | | | .742 | |  |  |
|  | DISTONYA LOWER LIMBS | .162 | | | .874 | |  |  |
|  | WALKING | -.674 | | | .512 | |  |  |
|  | TANDEM WALKING | -.464 | | | .650 | |  |  |
| RIGHT  GASTROCNEMIUS Walking | | | df | | | F | | Sig. |
| 1 |  | |  | | | 2.90 | | .047 |
|  |  | | 23 | | |  | |  |
| Walking P3 | | | df | | | F | | Sig. |
|  |  | |  | | | 1.064 | | .449^d^ |
|  |  | | 23 | | |  | |  |
| Walking | | t | | | Sig. | |  |  |
|  |  |  |  |  |  |  |  |  |
|  | CAG | 1.480 | | | .163 | |  |  |
|  | DURATION | **2.730** | | | **.017** | |  |  |
|  | UHDRSM | -.390 | | | .703 | |  |  |
|  | CHIOREA | -.840 | | | .416 | |  |  |
|  | CHLOREA LOWER LIMBS | .612 | | | .551 | |  |  |
|  | BRADIKINESIA | **2.457** | | | **.029** | |  |  |
|  | DISTONYA | -.968 | | | .351 | |  |  |
|  | DISTONYA LOWER LIMBS | 1.049 | | | .313 | |  |  |
|  | WALKING | .054 | | | .958 | |  |  |
|  | TANDEM WALKING | **2.324** | | | **.037** | |  |  |
| Walking P3 | | t | | | Sig. | |  |  |
|  |  |  |  |  |  |  |  |  |
| 1 | (Costante) | -.910 | | | .379 | |  |  |
|  | CAG | 1.493 | | | .159 | |  |  |
|  | DURATION | -.451 | | | .659 | |  |  |
|  | UHDRSM | .596 | | | .562 | |  |  |
|  | CHIOREA | -1.829 | | | .090 | |  |  |
|  | CHOREA LOWER LIMBS | .772 | | | .454 | |  |  |
|  | BRADIKINESIA | 1.626 | | | .128 | |  |  |
|  | DISTONYA | -.224 | | | .826 | |  |  |
|  | DISTONYA LOWER LIMBS | -.078 | | | .939 | |  |  |
|  | WALKING | -.511 | | | .618 | |  |  |
|  | TANDEM WALKING | -1.485 | | | .161 | |  |  |
| LEFT GASTRONEMIUS Walking | | | | df | | F | | Sig. |
|  |  | | |  | | .836 | | .605^d^ |
|  |  | | | 23 | |  | |  |
| Walking P3 | | | | df | | F | | Sig. |
|  |  | | |  | | 1.109 | | .422^d^ |
|  |  | | | 23 | |  | |  |
| Walking | | | | t | Sig. | |  |  |
|  |  |  |  |  |  |  |  |  |
|  | CAG | | | -.608 | .554 | |  |  |
|  | DURATION | | | 1.29 | .220 | |  |  |
|  | UHDRSM | | | .701 | .496 | |  |  |
|  | CHIOREA | | | -.956 | .357 | |  |  |
|  | CHOREA LOWER LIMBS | | | .547 | .594 | |  |  |
|  | BRADIKINESIA | | | -.923 | .373 | |  |  |
|  | DISTONYA | | | -.674 | .512 | |  |  |
|  | DISTONYA LOWER LIMBS | | | -.013 | .990 | |  |  |
|  | WALKING | | | -1.319 | .210 | |  |  |
|  | TANDEM WALKING | | | 1.214 | .246 | |  |  |
| Walking P3 | | | | t | Sig. | |  |  |
|  |  |  |  |  |  |  |  |  |
|  |  | | |  |  | |  |  |
|  | CAG | | | -.898 | .386 | |  |  |
|  | DURATION | | | .727 | .480 | |  |  |
|  | UHDRSM | | | 1.053 | .311 | |  |  |
|  | CHOREA | | | -1.483 | .162 | |  |  |
|  | CHOREA LOWER LIMBS | | | 1.052 | .312 | |  |  |
|  | BRADIKINESIA | | | -.531 | .604 | |  |  |
|  | DISTONYA | | | 1.182 | .102 | |  |  |
|  | DISTONYA LOWER LIMBS | | | .879 | .395 | |  |  |
|  | WALKING | | | -1.327 | .207 | |  |  |
|  | TANDEM WALKING | | | .917 | .376 | |  |  |

Table S5 Linear regression analysis between muscular recruitment during walking and P3 walking and main clinical features in HD patients. The total significance of the correlation was evaluated by ANOVA test, weighted for age. Results for single predictive variables are also reported.
